# Carotenoid Cleavage Dioxygenases: Identification, Expression, and Evolutionary Analysis of This Gene Family in Tobacco

**DOI:** 10.3390/ijms20225796

**Published:** 2019-11-18

**Authors:** Qianqian Zhou, Qingchang Li, Peng Li, Songtao Zhang, Che Liu, Jingjing Jin, Peijian Cao, Yongxia Yang

**Affiliations:** 1 National Tobacco Cultivation & Physiology & Biochemistry Research Centre, College of Tobacco Science, Henan Agricultural University, Zhengzhou 450002, China; zhouqianqian@stu.henau.edu.cn (Q.Z.); lipeng@stu.henau.edu.cn (P.L.); zhangsongzi@henau.edu.cn (S.Z.); liuche@stu.henau.edu.cn (C.L.); 2 Zhengzhou Tobacco Research Institute of CNTC, Zhengzhou 450002, China; liqc@ztri.com.cn (Q.L.); jjjscuedu@gmail.com (J.J.); pjcao@ztri.com.cn (P.C.)

**Keywords:** *CCDs*, *Nicotiana tabacum*, genome-wide identification, hormones and abiotic stresses, gene expression

## Abstract

Carotenoid cleavage dioxygenases (CCDs) selectively catalyze carotenoids, forming smaller apocarotenoids that are essential for the synthesis of apocarotenoid flavor, aroma volatiles, and phytohormone ABA/SLs, as well as responses to abiotic stresses. Here, 19, 11, and 10 *CCD* genes were identified in *Nicotiana tabacum*, *Nicotiana tomentosiformis*, and *Nicotiana sylvestris,* respectively. For this family, we systematically analyzed phylogeny, gene structure, conserved motifs, gene duplications, *cis*-elements, subcellular and chromosomal localization, miRNA-target sites, expression patterns with different treatments, and molecular evolution. *CCD* genes were classified into two subfamilies and nine groups. Gene structures, motifs, and tertiary structures showed similarities within the same groups. Subcellular localization analysis predicted that *CCD* family genes are cytoplasmic and plastid-localized, which was confirmed experimentally. Evolutionary analysis showed that purifying selection dominated the evolution of these genes. Meanwhile, seven positive sites were identified on the ancestor branch of the tobacco CCD subfamily. *Cis*-regulatory elements of the *CCD* promoters were mainly involved in light-responsiveness, hormone treatment, and physiological stress. Different *CCD* family genes were predominantly expressed separately in roots, flowers, seeds, and leaves and exhibited divergent expression patterns with different hormones (ABA, MeJA, IAA, SA) and abiotic (drought, cold, heat) stresses. This study provides a comprehensive overview of the *NtCCD* gene family and a foundation for future functional characterization of individual genes.

## 1. Introduction

Carotenoids, as C_40_ isoprenoid compounds, contribute to a series of important biological functions in vivo. As accessory photosynthetic pigments and general antioxidants, they play important roles in harvesting light and preventing photooxidation [1,2]. Carotenoids also serve as yellow, orange, and red pigments in plant leaves, flowers, fruits, and other tissues. The cleavage of carotenoids can produce various apocarotenoids, which are a diverse class of natural compounds that act as signaling molecules, related to hormones, volatiles, and signals, in many plants [3,4,5,6].

Carotenoid cleavage dioxygenase (CCD) is an important enzyme that catalyzes the conversion of various carotenoids into smaller apocarotenoids [7]. The *CCD* gene was first isolated from maize (originally named *Vp14*) [8]. Subsequently, *CCDs* were identified in many other plant species. To date, most *CCDs* have been classified into two subfamilies, namely carotenoid cleavage dioxygenases (*CCDs*) and 9-*cis*-epoxycarotenoid cleavage dioxygenases (*NCEDs*), including nine clades (*CCD1*, *4*, *7*, and *8;* and *NCED2*, *3*, *5*, *6*, and *9*), according to the classification method in *Arabidopsis thaliana* [9]. Additionally, carotenoid oxygenase genes have also been classified into five clades (namely *CCD1*, *4*, *7*, and *8* and *NCEDs*), based on phylogeny and gene function [4,10]. In addition to these, a new group, named *CCD-Like* (*CCDL*), was also identified in *Solanum lycopersicum* [11], *Fragaria vesca* [12], and *Malus domestica* [13].

Members of the *CCD* subfamily are involved in the synthesis of apocarotenoid flavor, aroma volatiles, and strigolactones (SLs), according to differences in substrates and regioselectivity [14,15]. They also take part in responses to various abiotic stresses, pigmentation, photosynthesis, and photoprotection [13,14]. Among these, CCD1 was reported to cleave C_40_-carotenoids and C_27_-apocarotenoids at the 9, 10 (9′, 10′) and 5, 6 (5′, 6′) double bonds in vivo to form C_13_/C_14_-compounds (such as α/β-ionone), which act as important aroma and flavor substances in many plants [16,17,18]. For example, *LeCCD1*, *PhCCD1*, *OfCCD1*, and *CmCCD1* contribute to the formation of the flavor volatile β-ionone in *F. vesca*, *Petunia hybrid*a, *Osmanthus fragrans* Lour, and *Cucumis melo* L in vivo, respectively [19,20,21]. CCD4 also functions in cleavage of the 9, 10 (9′, 10′) double bond, which has important roles in forming color, flavor, and aroma properties of flowers and fruits [22,23]. Further, *PpCCD4* was reported to determine the flesh color of peach [24], *CmCCD4a* contributes to the formation of the white color in *Chrysanthemum* [25], and decreases in *StCCD4* activity increase the content of carotenoids in potato tubers [26]. Moreover, the ablation of *OsCCD4a* via RNA interference was found to enhance carotenoid content in rice seed endosperms and leaves [27]. Additionally, *CsCCD4b* is highly responsive to salt and dehydration stresses in *Crocus sativus* [28]. *CCD7* and *CCD8* are mainly involved in the biosynthesis of SL. CCD7 can cleave 9-*cis*-β-carotene to produce C_27_ 9-*cis*-β-apo-10′-carotenal, which is then cleaved by CCD8 to generate C_18_ 13′-apo-β-carotenal, the precursor of phytohormone SLs [29,30]. Recently, studies have also found that SLs not only control the shoot branch but also regulate rhizosphere signaling and further affect plant architecture, as well as responses to biotic and abiotic stresses in plants [29,31,32].

Members of the NCED subfamily are enzymes that specifically cleave the 11, 12 (11′, 12′) double bond of 9-*cis*-epoxy carotenoids to form C_15_-xanthoxin, which is an important rate-limiting step in synthesis of the phytohormone abscisic acid (ABA) [33,34]. ABA exerts considerable physiological effects during plant developmental processes under normal and stress conditions, including seed dormancy, leaf senescence, and responses to various environmental stresses, among others [35,36]. In avocado, *PaNCED1* and *PaNCED3* encode proteins that can synthesize xanthoxin, the precursor of ABA, in vitro [34]. Further, the overexpression of *VaNCED1* and *VuNCED1* leads to the accumulation of ABA and enhances the expression of drought-responsive genes in grape and cowpea [37,38]. Of note, NCED3 was especially identified as the first indispensable rate-limiting enzyme required for the biosynthesis of ABA [8,39,40]. Our previous study revealed that RNA interference-mediated knockdown of *NtNCED3* could reduce drought tolerance and impair plant growth through ABA feedback regulation in *Nicotiana tabacum* [41]. In *A. thaliana*, *AtNCED3*, together with *AtNCED5*, is thought to contribute to the production of ABA, which then affects the regular growth of plants [39]. In addition, *AtNCED6* and *AtNCED9* are necessary for ABA biosynthesis during seed development [42]. These observations illustrate that *NCED*s regulate ABA synthesis, and then affect ABA-mediated signals involved in plant metabolic status and downstream stress responses.

Tobacco is not only an important model plant but also one of the most economically significant crops [40]. It is thus important to identify *NCED* and *CCD* gene family members in this species due to the importance of apocarotenoids and phytohormone ABA/SLs in plant development and stress responses. Although the *CCD* gene family has been researched in some other species, such as *Capsicum annuum* L [43], *F. vesca* [12], and *M. domestica* [13], among others, there have still been only a few similar studies conducted on tobacco to date. Here, we first identified and investigated the entire *CCD* gene family, including 19 *CCD* gene family members in allotetraploid *N. tabacum*, as well as 10 and 11 members in their two ancestors *N. sylvestris* and *N. tomentosiformis*, respectively. Then, we systematically analyzed the phylogeny, gene structure, conserved motifs, synteny, *cis*-elements, subcellular and chromosomal localization, and the molecular evolution of the *CCD* gene family. Three-dimensional (3D) structures of CCD proteins were also modeled. To obtain functional information on *CCDs*, their expression patterns were analyzed by quantitative real-time (qRT) PCR in different tissues and in response to different hormones (ABA, MeJA, IAA, SA) and abiotic (drought, cold, heat) stress treatments. Our results herein will provide a solid foundation for further functional studies on *CCD* genes in tobacco.

## 2. Results

### 2.1. Identification and Characterization of CCD

To identify *CCD* family members in the genomes of the tetraploid species *N. tabacum* (2*n* = 48) and its two progenitor diploid species, *N. tomentosiformis* (2*n* = 24) and *N. sylvestris* (2*n* = 24), the coding sequences of nine *Arabidopsis AtCCDs* were used as queries to search against the China Tobacco Genome Database V2.0 (http://www.tobaccodb.org/) (restricted) in the China Tobacco Gene Research Centre. Based on sequence similarity to *AtCCDs*, in total, 19, 11, and 10 *CCD* genes were retrieved from the genomes of *N. tabacum*, *N. tomentosiformis*, and *N. sylvestris*, respectively (Table 1). The *CCD* sequences of *S. lycopersicum* and *C. annuum* were also downloaded from Sol Genomics Network (https://solgenomics.net/). The *CCD* gene families in tobacco were named according to previous reports and their homologies to *Arabidopsis*, *S. lycopersicum,* and *C. annuum CCD* family genes in the phylogenetic tree, based on species’ abbreviations [9,11,43].

The basic information for *NtCCDs*, *NsyCCDs*, and *NtomCCDs*, including the gene IDs, lengths of genes, open reading frames (ORFs) and deduced amino acid sequences, molecular weights (Mws), isoelectric points (pIs), and chromosomal and subcellular locations are listed in Table 1. In *N. tabacum*, gene and ORF lengths ranged from 1795 to 10472 bp and 1470 to 1956 bp, respectively. The deduced protein lengths varied from 489 to 650 amino acids, the Mw from 55.733 to 73.341 kDa, and pI from 4.60 to 8.18. There were four *NtCCDs* genes on chromosome 6, and chromosomes 2, 7, 8, 9, 11, 13, 15, 16, 18, 20, 23, and 24 contained one each. The other three *NtCCD* genes remained on scaffolds.

In *N. sylvestris*, the gene lengths of *NsyCCDs* varied from 1518 to 13565 bp, and ORF lengths ranged from 1518 to 1953 bp. Regarding deduced NsyCCDs, protein lengths varied from 505 to 650 amino acids, Mw from 56.325 to 73.341 kDa, and pI from 5.56 to 7.60. Moreover, two *NsyCCD* genes remained on scaffolds, and eight genes remained on superscaffolds. For *NtomCCDs*, gene and ORF lengths ranged from 1752 to 10899 bp and from 1563 and 1953 bp, respectively. For deduced NtomCCDs, protein lengths varied from 523 to 650 amino acids, Mw from 59.238 to 73.176 kDa, and pI from 6.06 to 8.35. Three *NtoCCD* genes remained on scaffolds, and eight genes remained on superscaffolds.

### 2.2. Phylogenetic Analysis of CCD

**Figure 1:**
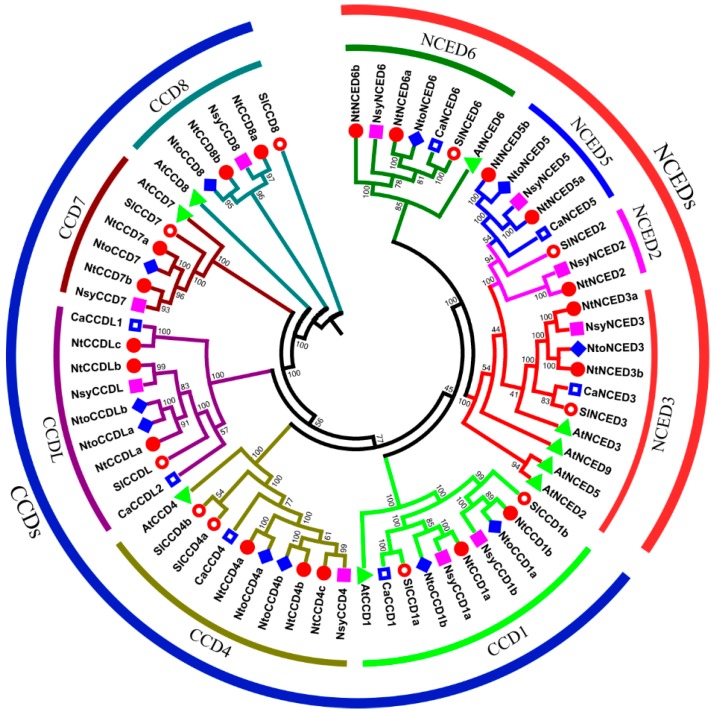
Phylogenetic analysis of CCD proteins from six species. Clustalx 6.0 was used to align the multiple sequences. Phylip3.69 was used to build the unrooted neighbor-joining (NJ) tree with 1000 bootstrap replicates. The CCD proteins of *Nicotiana tabacum*, *Nicotiana sylvestris*, *Nicotiana tomentosiformis*, *Arabidopsis thaliana*, *Solanum lycopersicum*, and *Capsicum annuum* were marked by red circle, pink square, light blue diamond, green triangle, red hollow circle, and blue hollow square, respectively. The protein/gene IDs in *Nicotiana tabacum*, *Nicotiana sylvestris*, and *Nicotiana tomentosiformis* are listed in Table 1, and the other three species, *Arabidopsis thaliana*, *Solanum lycopersicum*, and *Capsicum annuum*, are in Supplementary Table S1, respectively.

To better determine the evolutionary relationships among CCD proteins, an unrooted phylogenetic tree containing 66 CCD proteins from six species was constructed, using the neighbor-joining method in Phylip3.69 program (Figure 1). According to tree topology, all CCD proteins could be classified into two subfamilies (CCDs and NCEDs) and nine groups, named CCD1, CCD4, CCD7, CCD8, CCDL, NCED2, NCED3, NCED5, and NCED6, in accordance with the nomenclature of Tan [9], Wei [11], and Zhang [43] et al. Based on this grouping, for the CCD subfamily, two genes each from *N. tabacum*, *N. sylvestris*, and *N. tomentosiformis* belonged to the CCD1 group, whereas three NtCCDs, one NsyCCD, and two NtoCCDs fell into the CCD4 group. In addition, each of the CCD7 and CCD8 groups contained two NtCCDs, one NsyCCD, and one NtoCCD. Additionally, the CCDL group, which was recently proposed first in tomato [11], included three genes in *N. tabacum*, only one in *N. sylvestris*, and two in *N. tomentosiformis*, whereas none was identified in *Arabidopsis*, suggesting that CCDL proteins were probably expanded only within some species. Furthermore, phylogenetic relationships also revealed that CCD7, CCDL, and CCD8 groups might share the same ancestral gene, whereas the CCD1 and CCD4 groups were thought to have evolved more similarly. In addition, it could be speculated that the NCED subfamily had evolved from the CCD1 group. Moreover, only one NCED2 protein was found in *N. tabacum* and *N. sylvestris*. In addition, NCED3, NCED5, and NCED6 groups each contained two genes in *N. tabacum* and a unique NCED protein in *N. sylvestris* and *N. tomentosiformis*.

### 2.3. Gene Structures and Conserved Motifs Analysis of CCDs

**Figure 2:**
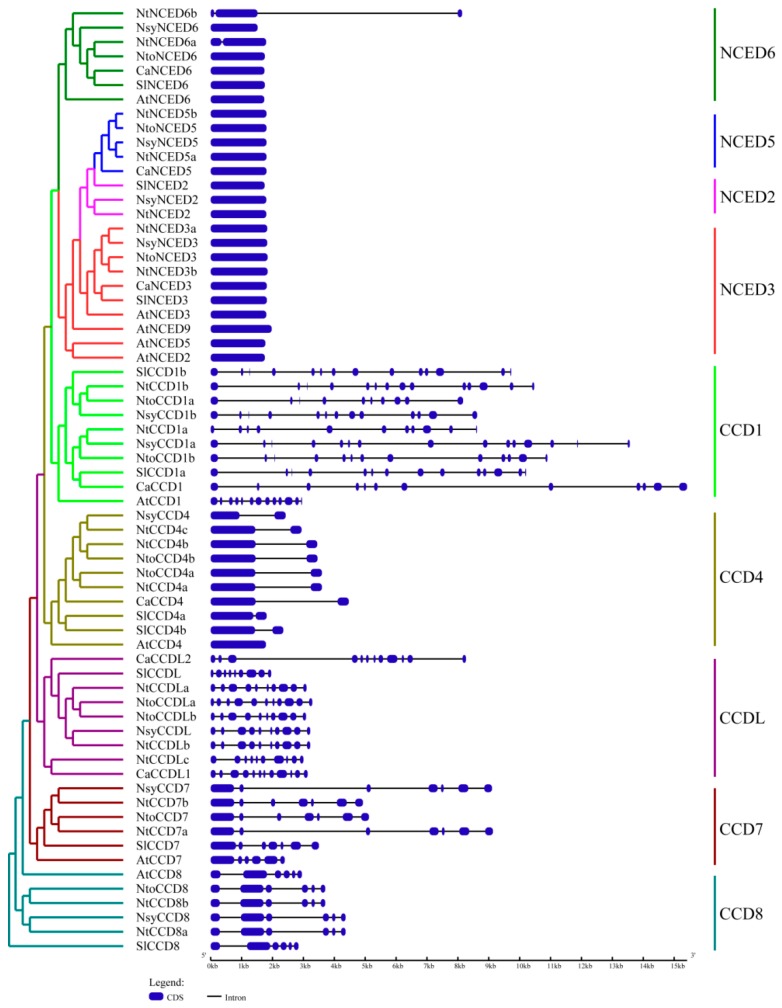
Schematic diagram of exon/intron structures of *CCD* genes from *Nicotiana tabacum*, *Nicotiana sylvestris*, *Nicotiana tomentosiformis*, *Arabidopsis thaliana*, *Solanum lycopersicum,* and *Capsicum annuum*. The unrooted neighbor-joining (NJ) evolutionary tree (left panel) was constructed by using Phylip3.69, with 1000 bootstrap replicates. The protein/gene IDs in *Nicotiana tabacum*, *Nicotiana sylvestris*, and *Nicotiana tomentosiformis* are listed in Table 1, and the other three species, *Arabidopsis thaliana*, *Solanum lycopersicum,* and *Capsicum annuum,* are in Supplementary Table S1, respectively. Exon–intron analyses of *CCD* genes were performed with GSDS 2.0 (right panel). Blue boxes and black lines correspond to exons (CDS) and introns, respectively.

To further obtain insights into the exon/intron structures of the *CCD* family, we compared their exon/intron arrangements by using GSDS online tools. As shown in Figure 2, the numbers of exons/introns were usually similar in the same group. All members of the *NCED* subfamily were intron-deficient (except *NtNCED6a* and *NtNCED6b*). Further, the *CCD1* group had the most introns, ranging from 9 to 14, and only one intron was found in the *CCD4* group. Five introns were present in the *CCD8* group. Moreover, the *CCDL* group contained 8–11 exons, whereas six introns were found in the *CCD7* group, except for *AtCCD*7, which had five introns.

**Figure 3:**
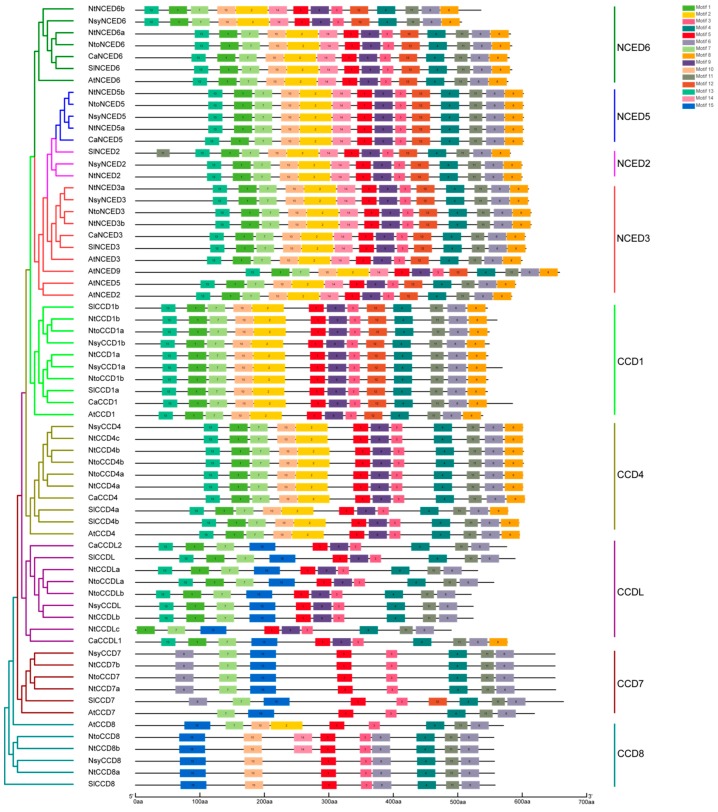
The conserved motifs of CCD proteins from *Nicotiana tabacum*, *Nicotiana sylvestris*, *Nicotiana tomentosiformis*, *Arabidopsis thaliana*, *Solanum lycopersicum,* and *Capsicum annuum*. In the left panel, the unrooted neighbor-joining (NJ) evolutionary tree was constructed by using Phylip3.69, with 1000 bootstrap replicates. The protein/gene IDs in *Nicotiana tabacum*, *Nicotiana sylvestris*, and *Nicotiana tomentosiformis* are listed in Table 1, and the other three species, *Arabidopsis thaliana*, *Solanum lycopersicum,* and *Capsicum annuum* are in Supplementary Table S1, respectively. In the right panel, different-color boxes represent different motifs.

All CCD proteins comprised an RPE65 domain (retinal pigment epithelial membrane protein) annotated based on the NCBI CDD database. To investigate the motif features of CCD proteins, 15 distinct motifs, representing part of the RPE65 domain, were identified with high e-values, using the MEME tool. The schematic distribution is shown in Figure 3. As expected, CCD proteins with close relationships showed similar motif compositions. Further, all groups of the CCD and NCED protein subfamilies contained motifs 3, 4, 5, 6, and 11, suggesting that these motifs might be important and responsible for their common functions. Of which, motifs 2, 5, 3, and 8 contained four conserved iron-ligating histidine (H) residues, and motifs 2, 4, and 11 contained conserved glutamate (E) or aspartate (D) residues required for activity. Exceptionally, some conserved residues were not found in these motifs of CCD7 and CCD8 and the CCDL group (Supplementary Table S2). In general, NCED subfamily proteins contained all 1–14 motifs, with motif 12 being specific to them. Motif 15 was absent in the NCED subfamily. Howbeit CCD proteins contained fewer motifs. Motif 14 and 15 were absent in the CCD1 group. In addition, CCD4 proteins lacked motifs 12, 14, and 15, and CCDL group proteins possessed 10 conserved motifs, with the absence of motifs 2, 8, 10, 12, and 14. Remarkably, NtCCDLc also lacked motif 13, and CaCCDL1 contained motif 8. Compared to those of the CCDL group, CCD7 group proteins lacked motifs 1, 9, and 13, and all CCD7 proteins had two motif 6s, except for AtCCD7. CCD8 harbored 8–9 motifs. In summary, these results indicated that each group of CCD subfamily proteins has different motif compositions, which further supports the topology of the phylogenetic tree.

### 2.4. Predicted Three-Dimensional (3D) Structures of NtCCDs

**Figure 4:**
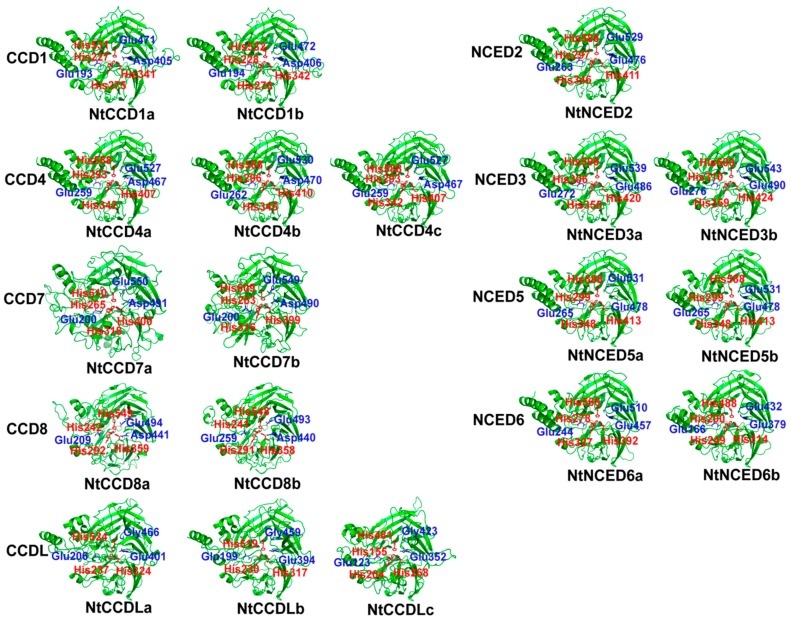
The predicted 3D structures of NtCCDs family proteins. The models were built with SWISS-MODEL and displayed with PyMOL software. The conserved four iron-ligating histidine (H) residues, and the conserved glutamates or aspartates (glycine in CCDL group) required for activity were marked with red and blue sticks and characters.

Tertiary structure prediction results indicated that CCD proteins are mainly comprised of random coils and β-strands (Figure 4). Moreover, proteins in the same group were found to be structurally similar, although there were some differences in the random coils. A comparable analysis of the 3D structures of different groups of CCDs revealed that they all have a conserved single loop composed of seven bladed β-sheets and one helix in these models, and that they also contained four conserved iron-ligating histidines and three glutamate or aspartate residues required for enzyme activity. The exception was the NtCCDL group, in which one of the glutamates was replaced with glycine in three NtCCDL proteins. Simultaneously, NtCCDLa and NtCCDLb lacked one conserved histidine (Supplementary Table S2). A comparison between different groups indicated that the structures of NtNCED subfamily proteins were more similar to those of the NtCCD1 and NtCCD4 groups. In contrast, NtCCD7, NtCCD8, and NtCCDL groups showed similar structures and possessed fewer or shorter α-helixes at N- and C-termini, which comprised the main differences between other CCD and NCED groups.

### 2.5. Chromosomal Locations and Gene Duplication Analysis of NtCCDs

**Figure 5:**
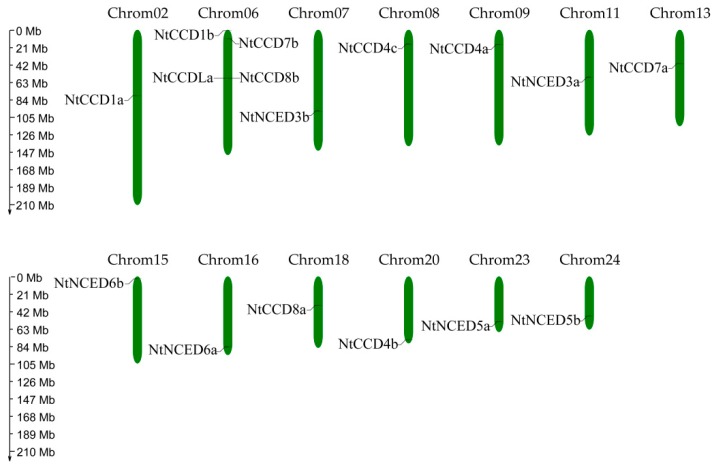
Chromosomal locations of *NtCCDs* genes on *Nicotiana tabacum* chromosome. The relative chromosome length scaled in megabases (Mb) is indicated on the left. The bars on chromosomes indicate the positions of the *NtCCDs* genes. The figure was generated and modified, using the MapGene2Chrom program.

We further determined the localizations of the *CCD* gene family on chromosomes of *N. tabacum*. Information from physical maps (including the numbers, lengths, and gene loci) of each *N. tabacum* chromosome was obtained from the China Tobacco Genome Database (V2.0). In general, 19 *NtCCDs* genes were unevenly distributed throughout 13 chromosomes and three genes were anchored to unattributed scaffolds (Figure 5, Table 1). No *NtCCDs* genes were mapped to the other 11 tobacco chromosomes. Four *NtCCDs* genes (*NtCCD1b*, *NtCCD7b*, *NtCCD8b*, and *NtCCDLa*) were located on chromosome 6, and 12 other chromosomes (2, 7, 8, 9, 11, 13, 15, 16, 18, 20, 23, 24) harbored only a single *NtCCD* gene each. In addition, due to a lack of physical map information for *N. sylvestris* and *N. tomentosiformis*, *NsyCCDs* and *NtoCCDs* could only be located on the scaffolds but not on the chromosomes of these species.

**Figure 6:**
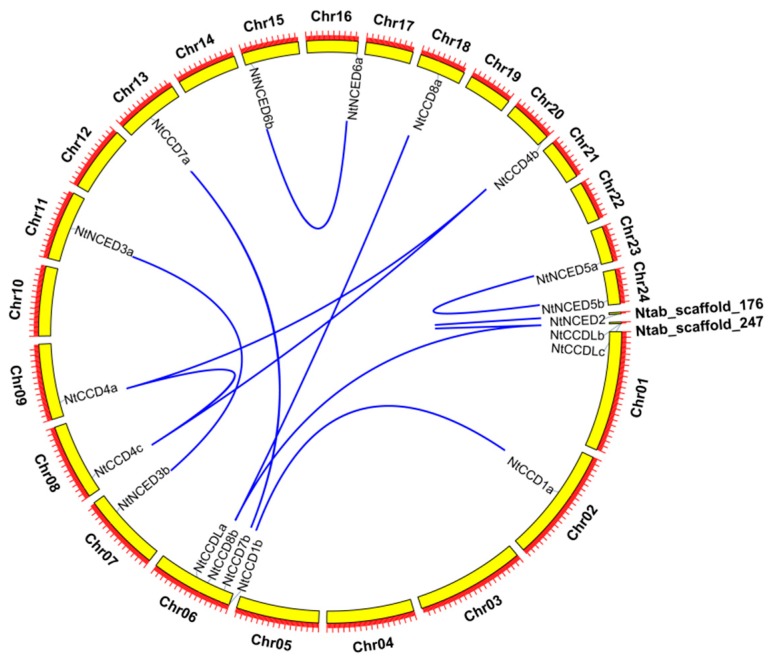
Gene duplications of the *NtCCDs* genes in *Nicotiana tabacum*. The relationships between *CCD* homologs were verified and visualized by using the Circos tool in TBtools software.

Gene duplication events occurred at least once during evolution in tetraploid tobacco. Gene duplication analysis might thus provide helpful information regarding the expansion of the *CCD* gene family. In our study, we analyzed the tandem duplication, as well as segmental duplication, of *NtCCD* genes. As shown in Figure 6, all of these paralogs (except for *NtCCD1b*, *NtCCD7b*, *NtCCD8b*, and *NtCCDLa*) were found to be located on different chromosomes, indicating that the homologous pairs were probably mainly produced by a whole-genome duplication (WGD) event and that segmental duplication was more important for the expansion of the *NtCCD* gene family. Adjacent homologous genes on the same chromosome are thought to be probably the result of a tandem duplication; however, *NtCCD1b*, *NtCCD7b*, *NtCCD8b*, and *NtCCDLa* did not share similar gene structures and motif compositions (Figure 2 and Figure 3). Moreover, according to the phylogenetic tree, they might have originated from an ancestor of tetraploid tobacco, namely *N. sylvestris* or *N. tomentosiformis*, suggesting that these genes might be preferential genes that were retained after WGD and subsequent frequent chromosomal arrangements.

### 2.6. Subcellular Localization of CCD Proteins

The N-terminal sorting signals and chloroplast transporter peptide (cTP) of CCD proteins were detected by using the online tools iPSORT and ChloroP Server program. Results showed that except for NsyNCED6, 15 CCD proteins in CCD4, NCED2, NCED5, and NCED6 groups of *N. tabacum*, *N. sylvestris*, and *N. tomentosiformis* had a chloroplast-targeting peptide at the N-termini, suggesting that these enzymes might be chloroplast-localized (Table 1). All CCD7 group proteins also had a mitochondrion-targeting peptide, and might be thus located at the mitochondrion. No sorting signal was detected in the remaining 21 CCD proteins. Then, we further investigated the subcellular localization of these CCD family members, using the online tools Plant-mPloc and ProtComp. The results showed that they were predicted to be cytoplasmic.

**Figure 7:**
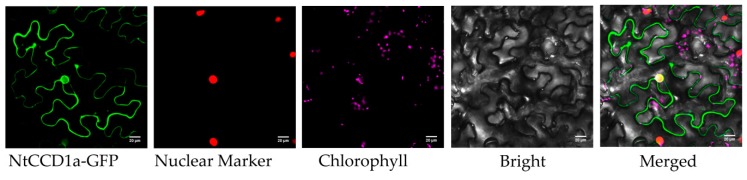
Subcellular localization of the NtCCD1a protein. NtCCD1a-GFP fusion proteins were transiently expressed in tobacco epidermal cells. Images were observed by laser confocal microscopy. Scale bar = 20 μm.

Using the transient expression methods induced by the *Agrobacterium*-mediated transformation, the cellular distributions of NtCCD1a proteins labeled by green fluorescent protein (GFP) were further investigated in tobacco leaves epidermal cells. In the leaves of the transformed control vector plant, the fluorescence of the GFP was detected in the cytoplasm and nucleus. After transient expression of NtCCD1a-GFP fusion proteins in tobacco leaves, the GFP signals were also merged with the nuclear localization signals (NLS) of the nucleus-anchored marker (Figure 7), suggesting that NtCCD1a was localized to both the cytoplasm and nucleus.

### 2.7. Cis-acting Elements Analysis of NtCCDs Promoters

**Figure 8:**
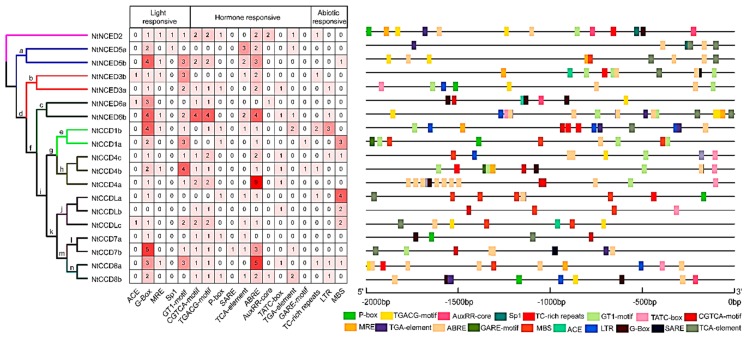
Promoter *cis*-elements analysis of *NtCCDs*. Left panel, the unrooted neighbor-joining (NJ) evolutionary tree. Middle panel, the number of *cis*-elements in *CCD* promoter. Right panel, the schematic distribution of *cis*-elements. Some *cis*-elements may overlap with others. Different *cis*-acting elements were displayed with different-color boxes. a–n: the foreground branch labeled in the molecular evolution analysis followed.

The promoter is an upstream segment of the gene, which plays important roles in the initiation of gene transcription. In this study, *cis*-acting elements of *NtCCD* promoters (2000 bp sequences upstream of ATG) were predicted using PlantCARE software. Finally, 18 potential *cis*-acting elements involved in light-responsiveness, hormone-responsiveness, and physiological stress (such as salt, drought, cold, heat, etc.) were identified in *NtCCD* promoters. The results indicating main elements and locations are as follows (Figure 8). Various light-responsive elements such as a GT1-motif, G-Box, ACE, and Sp1-binding site were found to exist in every *NtCCD* promoter. Especially, *NtCCD7b* promoters harbored five G-Box elements. The MRE is a MYB-binding site, which is present in some promoters and also involved in light responsiveness. The ABRE is involved in ABA responsiveness and can be found in most promoters. Among these, the majority has two or more ABREs. Specifically, the *NtCCD4a* promoter was found to contain nine ABREs based on tandem repeats, whereas the *NtCCD8a* promoter had five. Gibberellin-responsive elements include a P-box, GARE-motif, and TATC-box and appear in many promoters. MeJA-responsive elements, including the CGTCA-motif and TGACG-motif, were suggested to exist in most *NtCCD* promoters, except for those of *NtCCD1a*, *NtCCDLa*, *NtNCED3a*, and *NtNCED5a*. Salicylic acid responsive elements include a TCA-element (present in half of promoters) and SARE (only emerging in the *NtCCD7b* promoter). The AuxRR-core and TGA-element, involved in auxin responsiveness, was present in some promoters. TC-rich repeats as defense and stress responsive elements were found to exist in six promoters. Drought-inducibility elements (MBS) were found in some promoters, especially that of *NtCCDLa*, which has four MBSs. Six *NtCCD* promoters (*NtNCED3a*, *NtNCED6b*, *NtCCD1b*, *NtCCD4c*, *NtCCD8a*, and *NtCCD8b*) were identified has having a low-temperature responsive element (LTR). Overall, these results indicated that the expression of *NtCCD* family genes might be regulated by diverse *cis*-elements within the promoters related to light response, hormone and defense signal transduction, and abiotic stresses during tobacco growth.

### 2.8. Potential MicroRNA Target Sites of NtCCDs

MicroRNAs (miRNAs), usually small non-coding RNAs of approximately 22 nucleotides, regulate target genes related to plant development, responses to biotic/abiotic stresses, stress adaptation, and signal transduction via RNA silencing and post-transcriptional regulation mechanisms. The potential miRNA-target sites in *NtCCD* transcripts were searched, using psRNATarget database online. By maintaining a cut-off threshold of four as the search parameter, seven miRNAs from five families comprising target sites in five *NtCCD* family genes were identified, with an expectation score (E) ranging from 3.0 to 4 (Table 2). Among these, *NtCCD1a* and *NtCCDLc* contained target sites for multiple miRNAs, whereas *NtNCED3a*, *NtNCED3b*, *NtNCED6a*, and *NtCCD4a* comprised target sites for a single miRNA. As an important factor for target recognition, the numerical value of target accessibility (UPE) for the target site represents the energy required to bind and cleave target mRNA, and a lower energy means a greater possibility of interactions between miRNA and the target site. Here, UPE varied from 8.152 (nta-miR159) to 22.68 (nta-miR159 and nta-miR319b).

### 2.9. Molecular Evolution of NtCCDs

The Ka and Ks represent the nonsynonymous and synonymous substitution rate. The Ka/Ks (*ω*) value that is greater than, less than, and equal to 1 represents positive, negative (purify), and neutral selection, respectively [44]. To determine the possible selection pressures acting on *NtCCDs*, we analyzed whether positive selection existed during the evolution of *NtCCDs* by using the software EasyCodeML1.2. For the site model (Table 3), one-ratio model (M0), based on the average over all sites and branches, yielded an estimated *ω* = 0.09246. This result indicated that purifying selection dominated the evolution of tobacco *CCDs*. However, this model has its limitations. Then, the heterogeneous selective pressure among sites were tested, using different models. The discrete model (M3), which assumes variation among sites but no variation among branches, was applied to the sequences. Likelihood ratio tests (LRTs) of M0 against M3 indicated significant variation in selective pressure among the sites. Neutral model M1a assumes sites with two categories (0 < *ω*0 < 1 and *ω*1 = 1), and selection model M2a adds a third kind of site (*ω* > 1). LRTs of M1a against M2a didn’t find positive selection site. Model M7, M8, and M8a were used to estimate the spatial distribution of each site. LRTs of M7 against M8 and M8a against M8 both didn’t find a positive selection site. These site models found no evidence of a strong role of positive selection affecting the evolution of tobacco *CCD* genes, which implied that they were under purifying selection.

For the branch model (Table 4), 14 main clades of *NtCCDs* were defined as foreground branches. The results showed that all *ω* values were less than 1 for the 14 foreground branches (Figure 8), indicating that they were under purifying selection. We then applied the branch-site model, which accommodates heterogeneity among sites and can reflect divergent selective pressures. Parameter estimates under this model suggested that, when the ancestor branch of the tobacco *CCD* subfamily (labeled as i branch) was the foreground branch, a large set of sites (81.697%) evolved under strong purifying selection (*ω* = 0.063) and that a small set of sites (5.118%) evolved under neutral evolution (*ω* = 1); in addition, another small set of sites (13.185%) evolved under positive selective pressures (*ω* = 11.8) (Table 5). Using the Bayes empirical Bayes method, we identified seven codon sites with posterior probabilities ≥ 95% that evolved under positive selective pressure on the branch ancestral to the tobacco *CCD* subfamily (131K, 339I, 343A, 349G, 406F, 413Y, 533F, and NtCCD1a used as the amino acid reference). When the ancestor branch of *CCD1* and *CCD4* group genes (labeled as g branch) and the ancestor branch of *CCD7*, *CCD8*, and *CCDL* group genes (labeled as k branch) were the foreground branches, no codon sites with posterior probabilities ≥ 95% were identified under positive selective pressures.

### 2.10. Expression Patterns of NtCCDs in Different Tissues

**Figure 9:**
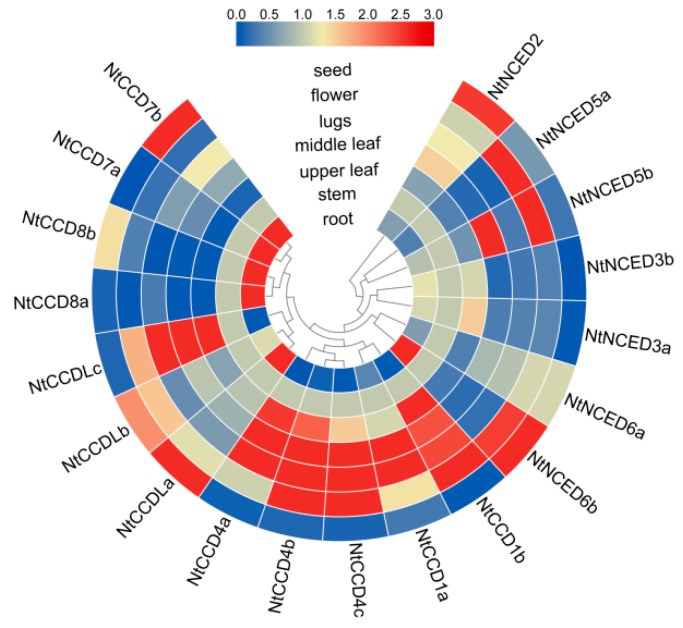
Heat map representation displaying the expression patterns of *NtCCDs* genes in different tissues in tobacco. The gene expression levels in the seven tissues were explored by qRT-PCR and visualized by TBtools software. The scale bar above indicates relative expression level.

To elucidate the roles of *NtCCD* genes, the tissue-specific expression levels of *CCD* genes were analyzed, using the qRT-PCR method. Seven samples including root, stem, upper leaf, middle leaf, lugs, flower, and seeds were collected. The results showed that most *NtCCD* genes were expressed in the upper leaf, middle leaf, and lugs. Five genes (*NtCCD1b*, *NtCCD4b*, *NtCCD4c*, *NtNCED5a*, and *NtNCED5b*) were highly expressed in the flower (Figure 9, Supplementary Table S3). Six genes (*NtCCD1a*, *NtCCD1b*, *NtCCD4a*, *NtCCD4b*, *NtCCD4c*, and *NtCCDLc*) were highly expressed in middle leaves and lugs, followed by the upper leaves and flower. Six genes (*NtCCD7a*, *NtCCD7b*, *NtCCD8a*, *NtCCD8b*, *NtNCED3a*, and *NtNCED3b*) showed the highest expression in roots. In contrast, four genes (*NtCCD7b*, *NtCCDLa*, *NtNCED2*, and *NtNCED6b*) were highly expressed in the seed. These diverse expression levels could suggest that *CCD* family genes have functional importance in different tissues.

### 2.11. Gene Expression Analysis of Responses to Hormone Treatments and Abiotic Stresses

**Figure 10:**
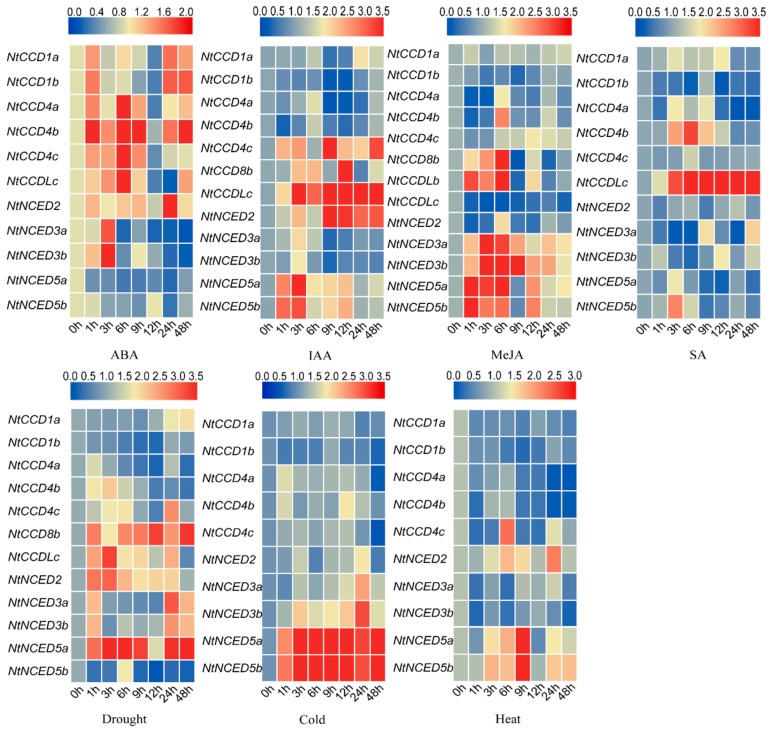
Heat map representation displaying the expression patterns of *NtCCDs* genes in the different hormone treatments (ABA, IAA, MeJA, SA) and abiotic stresses (drought, cold, heat). The gene expression levels were explored by qRT-PCR and visualized by TBtools software. The scale bars above indicate relative expression level.

To investigate the effects of plant hormones and abiotic stresses on *NtCCDs* genes, the expression patterns of *NtCCDs* induced by the four hormones (ABA, MeJA, IAA, SA) associated with plant abiotic stresses (drought, cold, heat) were analyzed by qRT-PCR (Figure 10, Supplementary Table S4a–g). Of note, we did not detect the expression of *NtCCD7a*, *NtCCD7b*, *NtCCD8a*, *NtCCDLa*, *NtNCED6a*, and *NtNCED6b* in response to these treatments. In ABA-treated samples, the expression of most genes exhibited two peaks, showing an increasing pattern after ABA treatment and plateauing at 3–9 h, subsequently decreasing following the next peak. However, *NtNCED5a* and *NtNCED5b* exhibited decreased expression levels. The expression of other genes was not obviously changed. With respect to IAA treatment, the expression of *NtCCD4c*, *NtCCD8b*, *NtCCDLc*, *NtNCED2*, *NtNCED5a*, and *NtNCED5b* was obviously induced but reached their highest levels at different points. The expression of six genes (*NtCCD8b*, *NtCCDLb*, *NtNCED3a*, *NtNCED3b*, *NtNCED5a*, and *NtNCED5b*) was significantly increased after MeJA treatment, whereas expression of the remaining genes changed only slightly. Moreover, only the expression of *NtCCD4c* and *NtCCDLc* was induced remarkably by SA treatment. Regarding abiotic stresses, drought could evidently induce the highest increase in the expression of *NtCCD8b*, *NtCCDLc*, *NtNCED3a*, *NtNCED3b*, and *NtNCED5a*, followed by that of *NtCCD4a*, *NtCCD4b*, and *NtCCD4c*, and most genes showed a bimodal trend. The expression of *NtNCED5a* and *NtNCED5b* was significantly induced by cold treatment and reached a peak level at 9 h. *NtNCED3a* and *NtNCED3b* were also affected, especially at 24 h. Finally, for high-temperature stress, most genes were downregulated; however, the expression of *NtCCD4c*, *NtNCED2*, *NtNCED5a*, and *NtNCED5b* was significantly upregulated, particularly at 6 or 9 h after treatment.

## 3. Discussion

### 3.1. Basic Characteristics of NtCCD Genes Family

Tobacco is both an important model and economically significant crop worldwide. CCDs play an essential role in the synthesis of apocarotenoid flavor, aroma volatiles, and phytohormone ABA/SLs, which are important for plant development, crop quality, and stress responses. Due to their great significance, in this study, we identified 19, 11, and 10 *CCD* gene family members in *N. tabacum*, *N. tomentosiformis*, and *N. sylvestris*, respectively. Most previous reports have demonstrated that the *CCD* gene family can be divided into *CCD* and *NCED* subfamilies, including five (*NCED* and *CCD1*, *4*, *7*, and *8*) or nine (*CCD1*, *4*, *7*, and *8;* and *NCED2*, *3*, *5*, *6*, and *9*) groups in *A. thaliana* [4,10]. Moreover, a new group, *CCDL*, was found only in some species [11]. According to the phylogenetic tree in this research, we identified and classified *CCD* gene families in tobacco into two subfamilies (*CCDs* and *NCEDs*) and nine groups (*CCD1*, *4*, *7*, *8*, and *L;* and *NCED2*, *3*, *5*, and *6*). No *NCED9* group was identified, because a corresponding ortholog from *Arabidopsis* was lacking. In addition, *NCED* subfamilies were found to be most closely related to *CCD1* and *CCD4* groups, whereas they had the most distant relationship with *CCD7*, *CCD8*, and *CCDL* groups. Tertiary structure prediction results also indicated that NtNCED proteins were structurally similar to NtCCD1 and NtCCD4 group proteins. In contrast, NtCCD7, NtCCD8, and NtCCDL were structurally more different, although all NtCCD family proteins had a conserved hydrophobic cavity composed mainly of β-sheets and a helix. Comprehensive analyses of chromosomal locations and gene duplication revealed that members in the same group were all located on different chromosomes, and, according to the phylogenetic tree, each originated from one ancestor of tetraploid tobacco, *N. sylvestris* or *N. tomentosiformis*. These results suggested that gene retention after WGD and chromosomal arrangements are responsible for expansion of the *CCD* gene family in tobacco. No tandem duplications were detected in this research; this outcome was inconsistent with previous reports on *M. domestica* [13].

Analyses of gene structures and motifs indicated that the numbers and positions of exons/introns and conserved motifs were usually similar in the same group. Intron numbers of all *NtCCD* genes ranged from 0 to 14, and motif numbers ranged from 7 to 14. The *CCD1* group had the most introns and these genes are reported to be involved in the production of aromatic volatile compounds and pigmentation [19,21]. Meanwhile, the *CCD4* group had only one intron, and it is involved in color formation, especially in flowers and fruits. *CCD7* and *CCD8* groups had five introns and harbored the fewest motifs, which may be involved in the biosynthesis of SL and related to branch growth development. Except for *NtNCED6a* and *NtNCED6b*, all *NCED* subfamily genes were intron-free but encoded more conserved motifs compared to that in the other groups, and this phenomenon is widespread in plants. Further, whether more motifs provide NCED proteins with more functions needs to be researched in the future. The absence of introns might be due to the loss of intron in the process of gene duplication. This intron-free structure of *NCED* genes is considered to be necessary for rapid and efficient synthesis of ABA and ABA-mediated stress response in plants, which is a result of a highly accurate posttranscriptional processing under stress [12]. The 3D structure prediction combined motif analysis and sequence alignment revealed that all CCD proteins contained four conserved iron-ligating histidine and three glutamate or aspartate residues required for enzyme activity, except for NtCCDLa and NtCCDLb. It is not known whether the lack of a conserved histidine in NtCCDLa and NtCCDLb affects enzyme activity and subsequently the function of the CCDL protein, considering that no CCDL functional or point mutations in these residues have been reported in related research. It would thus be helpful to clarify the function of the active site and *CCDL* genes, which could lead to novel insights for the *CCD* gene family.

### 3.2. Molecular Evolution of NtCCDs Genes in Tobacco

To gain new insight into the role of the *CCD* gene family, we evaluated possible selection pressures. The results of branch model analysis defined 14 main clades as foreground branches, which showed variable *ω* ratios among the groups; however, all *ω* values were less than 1, implying that purifying selection is the main driver of *CCD* evolution. Site models based on several paired comparisons (M0 vs. M3, M1a vs. M2a, M7 vs. M8, and M8a vs. M8) detected several sites of positive selection for all *CCD* groups. The results revealed that, although selective pressure varies among different *CCD* genes, there was no evidence of a strong role of positive selection in the evolution of *CCD* genes. This suggests that *CCD* sequences in tobacco plants are highly conserved, which might imply that *CCD* families have fulfilled certain requirements and that some of their functions are so important to tobacco that they have evolved with lower rates and cannot change optionally.

**Figure 11:**
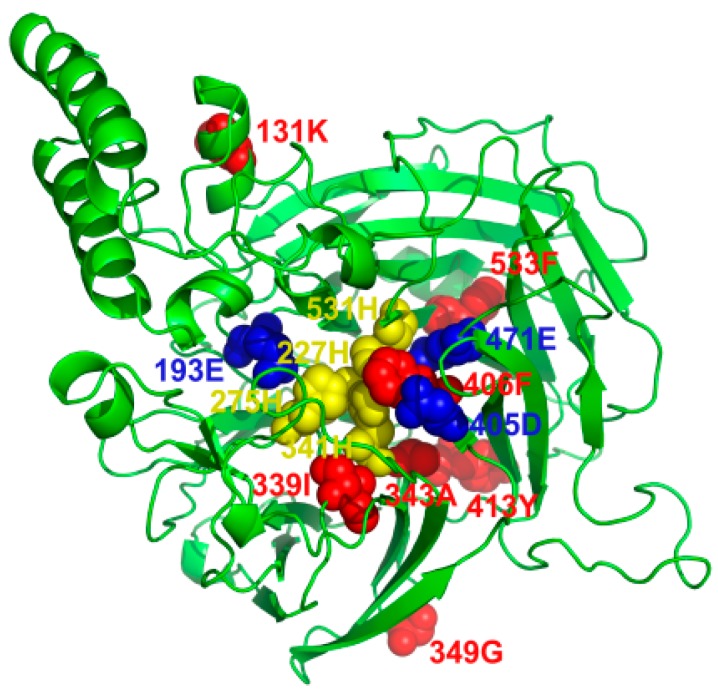
The predicted 3D structures and the positive sites of NtCCD1a (used as amino acid reference). The models were built with SWISS-MODEL and displayed with PyMOL software. The conserved four iron-ligating histidine (H) residues, and the conserved glutamates or aspartates (glycine in CCDL group) required for activity and positive sites were marked with yellow, blue, and red sphere, respectively.

For most genes, positive selection often occurs at only a few loci of a specific pedigree. To detect this type of local interpolation of natural selection signals, positive selection tests based on the branch and site model are greatly limited. The branch-site model, which allows the *ω* parameter to differ between branches and sites at the same time, can detect the sites that endure positive selection on the branches specified in advance. The prespecified branch is called the foreground branch, whereas the other branches are the background branches. The model assumes that the *ω* parameters are different between sites and that some *ω* parameters of the sites are subjected to positive selection on the foreground branches. In this study, we also applied the branch-site model. When the ancestor branch of the tobacco *CCD* subfamily was the foreground branch, most sites (81.697%) evolved under strong purifying selection, and a few sites (13.185%) evolved under positive selective pressures (*ω* = 11.8). After BEB analysis, seven codon sites with posterior probabilities ≥ 95% were identified as evolving under positive selective pressures (131K, 339I, 343A, 349G, 406F, 413Y, 533F, and NtCCD1a used as an amino acid reference). Combining protein structure and positive selection analyses can help to explain Darwinian selection on branches and sites. As shown in the structural model of NtCCD1a (Figure 11), 406F is located within the conserved hydrophobic cavity, surrounded by four conserved iron-ligating histidines and three glutamate or aspartate residues required for enzyme activity. Other positive sites are located around these conserved activity sites. Among these, 339I and 343A are located on motif 3, in addition to 406F and 413Y on motif 4, which are the two conserved motifs that were contained by all CCD and NCED subfamily proteins. According to common opinion, positive selection might drive diversification functions and possess biological significance during species evolution. This study will provide useful information and new insights into the functional divergence of *CCD* and *NCED* subfamilies, which is worth further investigation.

### 3.3. Regulation of NtCCD Genes in Tobacco

The *NtCCD* promoters were found to contain many potential elements involved in light-responsiveness (GT1-motif, G-Box, ACE, Sp1, MRE), hormone stress (ABRE, TCA-element, SARE, CGTCA-motif, TGACG-motif, CGTCA-motif, TGACG-motif, AuxRR-core, TGA-element), and physiological stress (TC-rich repeats, MBS, LTR). These results indicated that the *NtCCD* genes might be involved in these processes. We ultimately tested the expression of these genes upon exposure by several hormone treatments and abiotic stresses.

*CCD1* plays an important role in the production of apocarotenoid volatiles such as aroma and flavor substances [16,18]. Our results showed that *NtCCD1* is expressed mainly in leaves and flowers. The expression of *NtCCD1* can be induced by ABA and drought. *CCD4* is mainly involved in color-, flavor-, and aroma-formation in fruit, flowers, and leaves [22,23]. Similar to *NtCCD1*, *NtCCD4* was found to be highly responsive to ABA and dehydration stresses. This is consistent with a previous report [28]. *NtCCD4b* was also found to be markedly downregulated by MeJA and SA. Moreover, *NtCCD4c* was highly responsive to IAA. This illustrated that even genes of the same group might be affected by different factors and exhibit diverse functions. *CCD7* and *CCD8* are mainly involved in the biosynthesis of strigolactone, which controls the shoot branch and regulates rhizosphere signaling and response to stresses in plants [29,31,32]. Tissue-specific expression analysis showed that *NtCCD7* and *NtCCD8* exhibited the highest expression levels in roots. Moreover, no expression was detected in response to most treatments, except *NtCCD8b* was induced by IAA, MeJA, and drought. This phenomenon can be explained by the fact that these genes were found to be expressed mainly in roots, but we collected samples only from leaves. Regarding the *CCDL* group, no research has been performed on the function of these genes. *NtCCDLa* and *NtCCDLb* were found to be expressed mainly in roots and seeds, whereas *NtCCDLc* was mainly localized to leaves. *NtCCDLc* was strongly induced by ABA, IAA, SA, and drought, but was inhibited by MeJA. In contrast, *NtCCDLb* was strongly induced only by MeJA. Meanwhile, *NtCCDLa* was scarcely expressed in response to all treatments.

The *NCED* subfamily is believed to regulate an important rate-limiting step in the synthesis of ABA [33,34]. In this study, *NtNCED2*, *3*, *5*, and *6* were predominantly expressed in different tissues. *NtNCED2* was highly expressed in seeds and could be upregulated by ABA, IAA, drought, and heat. *NtNCED3* was expressed mainly in roots and upper leaves and was upregulated by ABA, MeJA, drought, and cold. Our previous research on *NtNCED3*, as well as a report on *A. thaliana*, demonstrated that *NCED3* is especially indispensable for the biosynthesis of ABA and can affect the growth of plants [39]. In contrast, ABA can be induced by abiotic stresses such as drought, cold, and heat, consistent with the upregulation expression of *NtNCED3*. *NtNCED5a* was found to be expressed mainly in flowers, whereas *NtNCED5b* was expressed in flowers and in middle leaves. IAA, MeJA, cold, and heat were also found to upregulate the expression of *NtNCED5a* and *NtNCED5b*. In addition, *NtNCED5a* was significantly induced by drought. Taken together, our results provide helpful information for further studies on the roles of *NtCCDs,* but more efforts are needed to clarify their diverse function.

We also searched the potential miRNA-target sites in the *NtCCD* transcripts. The identification of miRNA-target sites can provide clues into the biological functions of miRNAs/target genes during plant development and stress adaptation, and help to better understand functions and regulatory mechanisms. Seven miRNAs from five families in five *NtCCD* family genes were identified. Former research has shown that nta-miR6164a/b and nta-miR319/159 are related to the regulation of signal transduction, protein phosphorylation, cell differentiation, and virus infection [45,46]. Among plants, these miRNA families are usually highly conserved. However, this requires further verification in future studies.

## 4. Materials and Methods

### 4.1. Identification and Characterization of CCD

To perform genome-wide identification and obtain sequences of the *CCD* gene family in three *Nicotiana* species, nine published *AtCCD* sequences of *A. thaliana* were first downloaded from TAIR (http://www.arabidopsis.org). Then, using *AtCCD*s as queries, a blast search of tobacco genome sequences was performed. The *CCD* sequences of *N. tabacum*, *N. sylvestris*, *N. tomentosiformis*, and other species, including *S. lycopersicum* and *C. annuum,* were downloaded from China tobacco genome annotation project [restricted] and Sol Genomics Network (https://solgenomics.net/). BLASTN and BLASTP programs were carried out by using default parameters, respectively. To further confirm the *CCD* family members, the CCD proteins were predicted by Pfam (http://pfam.sanger.ac.uk/search) and SMART (http://smart.embl-heidelberg.de/) databases. The number of amino acids, Mws, and pIs of CCD proteins were calculated, using the online software ExPASy-ProtParam (https://web.expasy.org/protparam/).

### 4.2. Phylogenetic Analysis of CCD Proteins and Gene Nomenclature

The identified CCD proteins were aligned by using ClustalW. The phylogenetic tree of the CCD family was generated by using the neighbor-joining method, with 1000 bootstrap replicates in Phylip3.69 based on the Jones–Taylor–Thornton (JTT) matrix-based model. Genes that had been reported previously were named accordingly [9,11,43], and others were named according to their homologies to those of *Arabidopsis*, *S. lycopersicum*, and *C. annuum NCED* and *CCD* subfamily genes in the phylogenetic tree, based on the species abbreviation.

### 4.3. Gene Structures and Conserved-Motif Prediction

Exon–intron structures of *CCDs* were predicted by using the online software Gene Structure Display Server 2.0 (GSDS 2.0) (http://gsds.cbi.pku.edu.cn/, Peking University, Beijing, China) [47]. The conserved motifs in the CCD protein sequences were firstly identified by the online software MEME (Multiple Expectation Maximization for Motif Elicitation, http://meme-suite.org, developed and maintained by University of Nevada, Nevada, USA, and The University Of Queensland, Queensland, Australia, in addition to University of Washington, WA, USA) [48], using the following parameters: the optimum motif width was set from 6 to 200 and 15 as the maximum number of motifs. The discovered motifs were then annotated, using the Pfam program.

### 4.4. Three-Dimensional (3D) Structure Modeling of CCD Family Proteins

The 3D structures of CCD family proteins were computer-modeled by the SWISS-MODEL program. The models were displayed by PyMOL software (DeLano Scientific LLC, San Carlos, CA, USA).

### 4.5. Chromosomal Locations and Gene Duplication Analysis of CCD Genes in Tobacco

The physical positions of *NtCCDs* genes identified in tobacco were mapped to the chromosomes with the software MapGene2Chrome V2 (http://mg2c.iask.in/mg2c_v2.0/, Tobacco Research Institute of Chinese Academy of Agricultural Sciences, Qingdao, China), and they were used to describe the locations of *CCD* genes. For gene duplication analysis, the collinearity relationships between *CCD* homologs were verified and visualized using the Circos tool in TBtools software (South China Agricultural University, Guangzhou, China) [49].

### 4.6. Subcellular Localization

The ChloroP 1.1 Server program (http://www.cbs.dtu.dk/services/ChloroP/, DTU Health Tech, Copenhagen, Denmark) was used to predict the cTP of protein sequences. N-terminal sorting signals of the CCD proteins were also investigated, using the online software iPSORT (http://ipsort.hgc.jp/, University of Tokyo, Tokyo, Japan). The subcellular localizations of all CCD proteins were then predicted by using the online ExPASy tools Plant-mPloc (https://www.psort.org/, Simon Fraser University, BC, Canada) and ProtComp (http://linux1.softberry.com/all.htm, Softberry, Inc, Mount Kisco, NY, USA).

For experimental verification, the full length of *NtCCD1a* was amplified by specific primers, using the cDNA from young leaves of *Nicotiana tabacum* L. (*Honghua Dajinyuan*). The PCR products were linked with pBWA (V) HS-ccdb-GLosgfp vector to generate vector pBWA (V) HS-CCD1a-GFP fused with the green fluorescent protein (GFP) reporter gene. *NtCCD1a* fused with nuclear localization signal (NLS) protein marker was then cloned into pBWA (V) HS-ccdb-GLosgfp vector to generate the pBWA (V) HS-CCD1a-NLS fusion protein as a nuclear-anchored marker vector. The sequence of NLS was MDPKKKRKV in this study. Positive clones were confirmed by DNA sequencing and transformed into the *Agrobacterium tumefaciens* strain (GV3101) by electrotransformation, and then injected into the lower epidermis of tobacco leaves from 30-day-old plants. A corresponding empty vector was considered as the control. After two days of cultivation in low light, the localization of fluorescence signals was observed by using laser confocal microscope (Nikon C2-ER, Tokyo, Japan).

### 4.7. MiRNA Target Sites and Cis-Acting Regulatory Elements Prediction

*CCDs* were submitted to the online tool psRNATarget (http://plantgrn.noble.org/psRNATarget/, The Samuel Roberts Noble Foundation, Ardmore, OK, USA) for predicting miRNA’s target sites, using default settings and threshold. The promoter regions (2000 bp upstream of ATG) of *CCD* family genes were retrieved from the China tobacco database and were searched for identifying various *cis*-regulatory elements by the PlantCARE database (http://bioinformatics.psb.ugent.be/webtools/plantcare/html/).

### 4.8. Molecular Evolution of CCD Genes in Tobacco

The sequences of *NtCCD* genes were aligned based on codons, using ClustalW codons in the MEGA7 program (Tokyo Metropolitan University, Tokyo, Japan). To explore selective pressure between *NtCCD* gene sequences, after the removal of gaps, we performed strict statistical analysis, using the software EasyCodeML1.2 (Fujian Agriculture and Forestry University, Fuzhou, China) [50]. The site model, branch model, and branch-site model were used based on the Preset Mode. The LRT tests whether the selected model is significantly better than the null model.

### 4.9. Plant Materials and Treatments

*Nicotiana tabacum* L. (*Honghua Dajinyuan*) was used to analyze the expression of *NtCCD* genes. Tobacco seeds were germinated and cultured by using a floating seedling production system under normal conditions (28 °C, 14 h light, 10 h dark). Tobacco was grown in the same environment, and stress treatments were applied until three true leaves appeared. Different tissues (root, stem, upper leaf, middle leaf, lugs, flower, and seed) were also collected, in order to analyze *NtCCD* gene expression at the flowering stage.

For drought stress, seedlings were cultivated in a solution containing 20% (*w*/*v*) polyethylene (PEG) 6000 for 48 h. For low- and high-temperature treatment, seedlings were kept at 4 and 42 °C, respectively, in illuminated incubators, for 48 h. For hormone treatments, seedlings were sprayed with 100 µmol of ABA, MeJA, or indoleacetic acid (IAA) or 1 mmol of salicylic acid (SA) for 48 h. All true leaves were sampled at 0 (control), 1, 3, 6, 9, 12, 24, and 48 h, immediately frozen in liquid nitrogen, and stored at −80 °C, prior to RNA extraction. Three biological replicates were performed for each sample.

### 4.10. RNA Extraction, Reverse Transcription, and Quantitative Reverse-Transcription PCR

Total RNAs from different tissues above and under different stress treatments were extracted by using Plant Total RNA Isolation Kit (FOREGENE, Chengdu, China). HiScript II Q RT SuperMix for qPCR (+gDNA wiper) (Vazyme, Nanjing, China) was used for reverse transcription. The expression of *CCD* genes was tested by quantitative reverse-transcription PCR (qRT-PCR). The primers of *CCD* genes used for the qRT-PCR were shown in Table S5. The PCR reaction mixtures contained 1 μL of cDNA, 5 μL of AceQ Universal SYBR qPCR Master Mix (Vazyme, China), 0.2 μL of each of forward/reverse primers, and 3.6 μL of double-distilled nuclease-free water. Reactions were run in a 96-well optical plate (Eppendorf, Saxony, Germany) at 95 °C for 10 min, followed by 35 cycles of 95 °C for 10 s, 60 °C for 30 s, and melting curve (95 °C for 15 s, 60 °C for 15 s, melting for 20 m, 95 °C for 15 s). Each sample was analyzed with three technical replicates. Melting curve analysis was conducted in order to verify the specificity of the primers. The mRNA levels of *CCDs* were normalized to L25, and the method of 2^−ΔΔCt^ was used to calculate the expression of *CCD* genes [51].

## 5. Conclusions

In this study, 19 *NtCCD* gene family members in *N. tabacum*, 11 members in *N. tomentosiformis*, and 10 members in *N. sylvestris* were finally identified. According to the phylogenetic tree, they were classified into two subfamilies (*CCDs* and *NCEDs*) and nine groups (*CCD1*, *4*, *7*, *8*, and *L*; *NCED2*, *3*, *5*, and *6*). Moreover, members in the same group were all located on different chromosomes. Gene structures, conserved motifs, and tertiary structures were usually similar in the same group. Subcellular localization results predicted that *CCD* family genes are cytoplasmic and plastid-localized. Transient expression via *Agrobacterium*-mediated transformation confirmed this prediction. Evolutionary analysis further showed that the *NtCCD* genes were expanded, and no tandem duplication events during evolution were predicted. Analysis of molecular evolution demonstrated that purifying selection dominated the evolution of tobacco *CCD* families. However, seven positive sites were identified on the ancestor branch of the tobacco *CCD* subfamily. The expression of *NtCCD* genes was also found to be tissue-specific, and miRNA target-site prediction analysis identified seven miRNAs from five families in the five *NtCCD* family genes. *NtCCD* promoters were also found to contain many potential *cis*-acting elements involved in light-responsiveness, hormone stress, and physiological stress. The expression pattern of *NtCCDs* in response to several hormone treatments and abiotic stresses were detected. These results implied that *NtCCDs* play important roles in responses to different stresses, pigmentation, photosynthesis, and photoprotection. Our previous research showed that RNA interference of one of the *NtCCDs* family gene *NtNCED3* could impair plant growth, causing a stunted phenotype by inhibiting root development and reducing drought tolerance through ABA feedback regulation in *Nicotiana tabacum* [41]. This study will provide valuable clues for future functional studies of *CCD* genes, but more efforts are needed to determine their detailed effects of ABA/SLs and different carotenoid metabolites on tobacco growth, development, and stress tolerance.

## Figures and Tables

**Table 1:** The list of *CCD* gene essential information in tobacco.

Species	Gene Name	Gene ID	Gene Length (bp)	ORF Length (bp)	Amino Acid	MW (kDa)	PI	Chromosome	Protein Subcellular Localization
*Nicotiana tabacum*	*NtCCD1a*	Ntab0245430	8618	1641	546	61.080	6.01	Chr02	Cytoplasm
	*NtCCD1b*	Ntab0755880	10472	1683	560	62.932	6.51	Chr06	Cytoplasm
	*NtCCD4a*	Ntab0608760	3600	1803	600	65.858	7.16	Chr09	Chloroplast
	*NtCCD4b*	Ntab0943160	3449	1806	601	65.936	7.24	Chr20	Chloroplast
	*NtCCD4c*	Ntab0104800	2941	1803	600	65.709	6.44	Chr08	Chloroplast
	*NtCCD7a*	Ntab0292410	9135	1956	650	73.341	6.43	Chr13	Mitochondrion
	*NtCCD7b*	Ntab0552870	4928	1953	651	73.260	6.43	Chr06	Mitochondrion
	*NtCCD8a*	Ntab0551270	4366	1671	556	62.350	5.98	Chr18	Cytoplasm
	*NtCCD8b*	Ntab0369080	3707	1668	555	62.194	6.18	Chr06	Cytoplasm
	*NtCCDLa*	Ntab0667500	3102	1593	530	59.969	5.85	Chr06	Cytoplasm
	*NtCCDLb*	Ntab0429710	3221	1572	523	59.214	5.56	Ntab_scaffold_247	Cytoplasm
	*NtCCDLc*	Ntab0264020	3002	1470	489	55.733	6.34	Ntab_scaffold_247	Cytoplasm
	*NtNCED2*	Ntab0264020	1800	1800	599	66.844	7.60	Ntab_scaffold_176	Chloroplast
	*NtNCED3a*	Ntab0266320	1830	1830	609	67.754	7.28	Chr11	Cytoplasm
	*NtNCED3b*	Ntab0214800	1842	1842	613	68.191	7.66	Chr07	Cytoplasm
	*NtNCED5a*	Ntab0972510	1806	1806	601	67.018	6.12	Chr23	Chloroplast
	*NtNCED5b*	Ntab0027880	1806	1806	601	67.134	6.40	Chr24	Chloroplast
	*NtNCED6a*	Ntab0557140	1795	1746	581	64.607	8.18	Chr16	Chloroplast
	*NtNCED6b*	Ntab0434870	8137	1608	535	60.419	4.60	Chr15	Cytoplasm
*Nicotiana sylvestris*	*NsyCCD1a*	Nsyl0144600	13565	1707	568	63.685	6.49	Nsyl_scaffold_1522	Cytoplasm
	*NsyCCD1b*	Nsyl0177010	8624	1647	548	61.316	6.33	Nsyl_superscaffold_202	Cytoplasm
	*NsyCCD4*	Nsyl0462250	2426	1803	600	65.691	6.62	Nsyl_superscaffold_41	Chloroplast
	*NsyCCD7*	Nsyl0304670	9105	1953	650	73.341	6.43	Nsyl_superscaffold_23	Mitochondrion
	*NsyCCD8*	Nsyl0145940	4366	1671	556	62.350	5.98	Nsyl_superscaffold_190	Cytoplasm
	*NsyCCDL*	Nsyl0401980	3221	1572	523	59.214	5.56	Nsyl_superscaffold_155	Cytoplasm
	*NsyNCED2*	Nsyl0071360	1800	1800	599	66.844	7.60	Nsyl_superscaffold_251	Chloroplast
	*NsyNCED3*	Nsyl0483670	1830	1830	609	67.754	7.28	Nsyl_superscaffold_179	Cytoplasm
	*NsyNCED5*	Nsyl0074010	1806	1806	601	67.020	6.12	Nsyl_scaffold_1118	Chloroplast
	*NsyNCED6*	Nsyl0472000	1518	1518	505	56.325	5.83	Nsyl_superscaffold_26	Cytoplasm
*Nicotiana tomentosiformis*	*NtoCCD1a*	Ntom0051870	8169	1647	548	61.657	6.27	Ntom_scaffold_148	Cytoplasm
	*NtoCCD1b*	Ntom0051880	10899	1641	546	61.138	6.06	Ntom_scaffold_14	Cytoplasm
	*NtoCCD4a*	Ntom0284420	3600	1803	600	65.858	7.16	Ntom_scaffold_554	Chloroplast
	*NtoCCD4b*	Ntom0290610	3458	1806	601	65.940	6.58	Ntom_superscaffold_38	Chloroplast
	*NtoCCD7*	Ntom0214430	5121	1953	650	73.176	6.43	Ntom_scaffold_374	Mitochondrion
	*NtoCCD8*	Ntom0326520	3707	1668	555	62.183	6.30	Ntom_scaffold_709	Cytoplasm
	*NtoCCDLa*	Ntom0006270	3289	1668	555	62.702	6.34	Ntom_scaffold_103	Chloroplast
	*NtoCCDLb*	Ntom0326460	3086	1563	523	59.238	6.23	Ntom_scaffold_709	Cytoplasm
	*NtoNCED3*	Ntom0303590	1842	1842	613	68.177	7.66	Ntom_scaffold_600	Cytoplasm
	*NtoNCED5*	Ntom0232040	1806	1806	601	67.094	6.37	Ntom_superscaffold_17	Chloroplast
	*NtoNCED6*	Ntom0304970	1752	1752	583	64.887	8.35	Ntom_superscaffold_63	Chloroplast

**Table 2:** The potential miRNA target sites in *NtCCD*s transcripts.

miRNA_Acc.	Target_Acc.	Expectation	UPE	miRNA Length	Target Start	Target End	Inhibition	Multiplicity
nta-miR319a	*NtCCDLc*	3	21.947	21	1266	1286	Cleavage	1
nta-miR319b	*NtCCDLc*	3	22.68	21	1265	1285	Cleavage	1
nta-miR159	*NtCCDLc*	3	22.68	21	1265	1285	Cleavage	1
nta-miR6150	*NtNCED3a*	3.5	10	22	1433	1454	Cleavage	1
nta-miR6150	*NtNCED3b*	3.5	11.413	22	1445	1466	Cleavage	1
nta-miR6150	*NtNCED6a*	4	21.187	22	1112	1134	Translation	1
nta-miR6148b	*NtCCD1a*	4	14.183	21	645	665	Translation	1
nta-miR6164a	*NtCCD1a*	4	18.268	21	356	376	Translation	1
nta-miR6164b	*NtCCD1a*	4	18.268	21	356	376	Translation	1
nta-miR159	*NtCCD4a*	4	8.152	21	324	344	Translation	1

**Table 3:** Parameter estimates and likelihood values for *NtCCD*s using the site model.

Model	Np	Ln L	Estimates of Parameters	RT Pairs	LRT *p*-Value	Positive Sites
M0: one ratio	38	−13,579.23	*ω*0 = 0.09246			
M3: discrete	42	−13,413.93	*p*0 = 0.14105, *p*1 = 0.62465, *p*2 = 0.23430,	M0 vs. M3	0.00	None
			*ω*0 = 0.01164, *ω*1 = 0.06928, *ω*2 = 0.25309			
M2a: selection	41	−13,554.18	*p*0 = 0.935245, *p*1 = 0.03719, *p*2 = 0.02757,			
			*ω*0 = 0.08829, *ω*1 = 1.00000, *ω*2 = 1.00000			
M1a: neutral	39	−13,554.18	*p*0 = 0.93524, *p*1 = 0.06476,	M1a vs. M2a	1.00	None
			*ω*0 = 0.08829, *ω*1 = 1.00000			
M8: beta and *ω* > 1	41	−13,430.72	*p*0 = 0.99883, *p* = 1.12281, *q* = 9.15782,	M7 vs. M8	0.9839	None
			*p*1 = 0.00117, *ω* = 1.00000			
M7: beta	39	−13,430.73	*p* = 1.11747, *q* = 9.06492			
M8a: beta and *ω* = 1	40	−13,419.70	*p*0 = 0.99999, *p* = 1.35368, *q* = 11.54435,	M8a vs. M8	0.000002674	None
			*p*1 = 0.0000, *ω* = 1.00000			

**Table 4:** Parameter estimates and likelihood values for *NtCCD*s using the branch model.

Model	Np	Ln L	Estimates of Parameters	LRT *p*-Value	Positive Sites (BEB)
Model 0	38	−13579.23	*ω* = 0.09246		
Two ratio branch a	39	−13575.32	*ω*0 = 0.10005, *ω*1 = 0.04060	0.005	None
Two ratio branch b	39	−13564.78	*ω*0 = 0.10374, *ω*1 = 0.00918	0.000	None
Two ratio branch c	39	−13574.47	*ω*0 = 0.09358, *ω*1 = 0.00164	0.002	None
Two ratio branch d	39	−13574.37	*ω*0 = 0.09763, *ω*1 = 0.02200	0.002	None
Two ratio branch e	39	−13574.07	*ω*0 = 0.09443, *ω*1 = 0.00421	0.001	None
Two ratio branch f	39	−13578.74	*ω*0 = 0.09401, *ω*1 = 0.04312	0.321	None
Two ratio branch g	39	−13577.44	*ω*0 = 0.09348, *ω*1 = 0.00024	0.059	None
Two ratio branch h	39	−13574.78	*ω*0 = 0.09439, *ω*1 = 0.00432	0.003	None
Two ratio branch i	39	−13572.92	*ω*0 = 0.09489, *ω*1 = 0.00216	0.00	None
Two ratio branch j	39	−13579.19	*ω*0 = 0.09241, *ω*1 = 0.12516	0.772	None
Two ratio branch k	39	−13578.89	*ω*0 = 0.09278, *ω*1 = 0.00154	0.409	None
Two ratio branch l	39	−13579.14	*ω*0 = 0.09277, *ω*1 = 0.05689	0.662	None
Two ratio branch m	39	−13578.69	*ω*0 = 0.09311, *ω*1 = 0.00201	0.297	None
Two ratio branch n	39	−13576.50	*ω*0 = 0.09367, *ω*1 = 0.00833	0.019	None

**Table 5:** Parameter estimates and likelihood values for *NtCCD*s, using the branch-site model.

Model	Np	Ln L	Estimates of Parameters	LRT *p*-Value	Positive Sites (BEB)
Model A (Branch g)	41	−13551.77	*p*0 = 0.90022, *p*1 = 0.06108, *p*2a = 0.03624, *p*2b = 0.00246, *ω*0 = 0.08803, *ω*1 = 1.00000, *ω*2a = 999.00000, *ω*2b = 999.00000	0.03	None
Model A Null (Branch g)	40	−13554.00	*p*0 = 0.86534, *p*1 = 0.05982, *p*2a = 0.07000, *p*2b =0.00484, *ω*0 = 0.08808, *ω*1 = 1.00000, *ω*2a = 1.00000, *ω*2b = 1.00000		Not Allowed
Model A (Branch i)	41	−13545.47	*p*0 = 0.81697, *p*1 = 0.05118, *p*2a = 0.12408, *p*2b = 0.00777, *ω*0 = 0.08849, *ω*1 = 1.00000, *ω*2a = 11.81766, *ω*2b = 11.81766	0.01	131K *, 339I **, 343A *, 349G *, 406F *, 413Y *, 533F *
Model A Null (Branch i)	40	−13548.71	*p*0 = 0.79861, *p*1 = 0.05219, *p*2a = 0.14005, *p*2b = 0.00915, *ω*0 = 0.0867, *ω*1 = 1.00000, *ω*2a = 1.00000, *ω*2b = 1.00000		Not Allowed
Model A (Branch k)	41	−13545.83	*p*0 = 0.79074, *p*1 = 0.04952, *p*2a = 0.15032, *p*2b = 0.00941, *ω*0 = 0.08852, *ω*1 = 1.00000, *ω*2a = 999.00000, *ω*2b = 999.00000	0.0009	None
Model A Null (Branch g)	40	−13551.29	*p*0 = 0.77180, *p*1 = 0.05151, *p*2a = 0.16563, *p*2b = 0.01105, *ω*0 = 0.08705, *ω*1 = 1.00000, *ω*2a = 1.00000, *ω*2b = 1.00000		Not Allowed

* *p* < 0.05, ** *p* < 0.01. BEB: Bayes empirical Bayes.

## Supplementary Materials

Supplementary materials can be found at https://www.mdpi.com/1422-0067/20/22/5796/s1.

## Abbreviations

CCD	Carotenoid cleavage dioxygenase
NCED	9-*Cis*-epoxycarotenoid dioxygenase
ABA	Abscisic acid
SL	Strigolactone
IAA	Indole-3-acetic acid
SA	Salicylic acid
Mw	Molecular weight
pI	Isoelectric point
ACE	*Cis*-acting element involved in light responsiveness
G-Box	*Cis*-acting regulatory element involved in light responsiveness
MRE	MYB binding site involved in light responsiveness
Sp1	Light responsive element
GT1-motif	Light responsive element
CGTCA-motif	*Cis*-acting regulatory element involved in the MeJA-responsiveness
TGACG-motif	*Cis*-acting regulatory element involved in the MeJA-responsiveness
P-box	Gibberellin-responsive element
SARE	*Cis*-acting element involved in salicylic acid responsiveness
TCA-element	*Cis*-acting element involved in salicylic acid responsiveness
ABRE	*Cis*-acting element involved in the abscisic acid responsiveness
AuxRR-core	*Cis*-acting regulatory element involved in auxin responsiveness
TATC-box	*Cis*-acting element involved in gibberellin-responsiveness
TGA-element	Auxin-responsive element
GARE-motif	Gibberellin-responsive element
TC-rich repeats	*Cis*-acting element involved in defense and stress responsiveness
LTR	*Cis*-acting element involved in low-temperature responsiveness
MBS	MYB binding site involved in drought-inducibility
