# Supplementary material for: Carotenoid Cleavage Dioxygenases: Identification, Expression, and Evolutionary Analysis of This Gene Family in Tobacco
Source: Int J Mol Sci. 2019 Nov 18;20(22):5796. doi: 10.3390/ijms20225796 (PMC6888377; doi:10.3390/ijms20225796)
Supplement: Supplementary file 1 [file ijms-20-05796-s001.pdf]

**Table S1** The protein / Gene ID of predicted carotenoid dioxygenase genes from the other 3 species, including *Arabidopsis thaliana* (At), *Solanum lycopersicum* (Sl) and *Capsicum annuum* (Ca).

| Gene Name | Protein / Gene ID | Gene Name | Protein / Gene ID | Gene Name | Protein / Gene ID |
|---|---|---|---|---|---|
| *AtCCD1* | AT3G63520 | *SlCCD1a* | Solyc01g087250 | *CaCCD1* | CA01g20280 |
| *AtCCD4* | AT4G19170 | *SlCCD1b* | Solyc01g087260 | *CaCCD4* | CA01g08910 |
| *AtCCD7* | AT2G44990 | *SlCCD4a* | Solyc08g075480 | *CaCCDL1* | CA08g04710 |
| *AtCCD8* | AT4G32810 | *SlCCD4b* | Solyc08g075490 | *CaCCDL2* | CA11g20400 |
| *AtNCED2* | AT4G18350 | *SlCCD7* | Solyc01g090660 | *CaNCED3* | CA07g16140 |
| *AtNCED3* | AT3G14440 | *SlCCD8* | Solyc08g066650 | *CaNCED5* | CA08g03620 |
| *AtNCED5* | AT1G30100 | *SlCCDL* | Solyc08g066720 | *CaNCED6* | CA05g17080 |
| *AtNCED6* | AT3G24220 | *SlNCED2* | Solyc08g016720 | | |
| *AtNCED9* | AT1G78390 | *SlNCED3* | Sl07g056570 | | |
| | | *SlNCED6* | Solyc05g053530 | | |

**Table S2** Conserved residues of NtCCDs

| | E193 | H227 | H275 | H341 | D/E405 | E471 | H531 |
|---|---|---|---|---|---|---|---|
| NtCCD1a | motif 2 | motif 2 | motif 5 | motif 3 | motif 4 | motif 11 | motif 8 |
| NtCCD1b | motif 2 | motif 2 | motif 5 | motif 3 | motif 4 | motif 11 | motif 8 |
| NtCCD4a | motif 2 | motif 2 | motif 5 | motif 3 | motif 4 | motif 11 | motif 8 |
| NtCCD4b | motif 2 | motif 2 | motif 5 | motif 3 | motif 4 | motif 11 | motif 8 |
| NtCCD4c | motif 2 | motif 2 | motif 5 | motif 3 | motif 4 | motif 11 | motif 8 |
| NtCCD7a | motif 15 | | | motif 3 | motif 4 | motif 11 | |
| NtCCD7b | motif 15 | | | motif 3 | motif 4 | motif 11 | |
| NtCCD8a | | | motif 5 | motif 3 | motif 4 | motif 11 | |
| NtCCD8b | | | motif 5 | motif 3 | motif 4 | motif 11 | |
| NtCCDLa | motif 15 | | | motif 3 | motif 4 | motif 11 | |
| NtCCDLb | motif 15 | | | motif 3 | motif 4 | motif 11 | |
| NtCCDLc | motif 15 | | | motif 3 | motif 4 | motif 11 | |
| NtNCED2 | motif 2 | motif 2 | motif 5 | motif 3 | motif 4 | motif 11 | motif 8 |
| NtNCED3a | motif 2 | motif 2 | motif 5 | motif 3 | motif 4 | motif 11 | motif 8 |
| NtNCED3b | motif 2 | motif 2 | motif 5 | motif 3 | motif 4 | motif 11 | motif 8 |
| NtNCED5a | motif 2 | motif 2 | motif 5 | motif 3 | motif 4 | motif 11 | motif 8 |
| NtNCED5b | motif 2 | motif 2 | motif 5 | motif 3 | motif 4 | motif 11 | motif 8 |
| NtNCED6a | motif 2 | motif 2 | motif 5 | motif 3 | motif 4 | motif 11 | motif 8 |
| NtNCED6b | motif 2 | motif 2 | motif 5 | motif 3 | motif 4 | motif 11 | motif 8 |

**Table S3.** The expression patterns of *NtCCDs* genes in different tissues

| Gene name | Root | Stem | Upper Leaf | Middle Leaf | Lugs | Flower | Seed |
|---|---|---|---|---|---|---|---|
| *NtCCD1a* | 0.32 ± 0.01** | 1.00 ± 0.02 | 1.15 ± 0.02** | 11.77 ± 0.09** | 10.90 ± 0.11** | 1.57 ± 0.0** | 0.22 ± 0.00** |
| *NtCCD1b* | 0.02 ± 0.01** | 1.00 ± 0.01 | 8.61 ± 0.19** | 4.07 ± 0.14** | 4.28 ± 0.06** | 22.94 ± 0.16** | 0.02 ± 0.05** |
| *NtCCD4a* | 0.00 ± 0.00 | 1.00 ± 0.00 | 4.62 ± 0.04** | 174.85 ± 2.42** | 111.69 ± 0.45** | 1.09 ± 0.32 | 0.06 ± 0.01 |
| *NtCCD4b* | 0.07 ± 0.01 | 1.00 ± 0.21 | 3.71 ± 0.07** | 108.13 ± 1.14** | 82.71 ± 0.75** | 10.51 ± 0.04** | 0.10 ± 0.11 |
| *NtCCD4c* | 0.01 ± 0.00 | 1.00 ± 0.03 | 1.86 ± 0.15 | 6.23 ± 0.18 | 6.46 ± 0.70 | 91.53 ± 7.20** | 0.08 ± 0.00 |
| *NtCCD7a* | 89.01 ± 6.64** | 1.01 ± 0.13 | 0.03 ± 0.00 | 0.38 ± 0.10 | 0.53 ± 0.14 | 0.18 ± 0.02 | 0.00 ± 0.00 |
| *NtCCD7b* | 378.39 ± 10.99** | 1.00 ± 0.05 | 0.09 ± 0.01 | 0.63 ± 0.16 | 1.44 ± 0.44 | 0.15 ± 0.10 | 76.02 ± 17.00** |
| *NtCCD8a* | 5.78 ± 0.49** | 1.00 ± 0.03 | 0.01 ± 0.00** | 0.01 ± 0.00** | 0.23 ± 0.02** | 0.01 ± 0.0**0 | 0.08 ± 0.00** |
| *NtCCD8b* | 6.73 ± 0.27** | 1.00 ± 0.10 | 0.01 ± 0.00** | 0.02 ± 0.00** | 0.00 ± 0.00** | 0.27 ± 0.01** | 1.59 ± 0.03** |
| *NtCCDLa* | 84.30 ± 3.51** | 1.00 ± 0.04 | 0.91 ± 0.10 | 0.72 ± 0.15 | 0.49 ± 0.08 | 1.28 ± 0.29 | 99.29 ± 2.92** |
| *NtCCDLb* | 1.22 ± 0.08 | 1.00 ± 0.05 | 0.57 ± 0.03** | 0.94 ± 0.16 | 0.38 ± 0.09** | 1.92 ± 0.1**3 | 2.78 ± 0.35** |
| *NtCCDLc* | 0.01 ± 0.00 | 1.00 ± 0.06 | 10.74 ± 0.53** | 13.60 ± 1.93** | 10.13 ± 0.34** | 2.24 ± 0.13 | 0.10 ± 0.00 |
| *NtNCED2* | 0.53 ± 0.01** | 1.00 ± 0.07 | 0.56 ± 0.03** | 1.78 ± 0.27** | 1.45 ± 0.12** | 1.10 ± 0.10 | 4.61 ± 0.35** |
| *NtNCED3a* | 1.17 ± 0.04** | 1.00 ± 0.01 | 1.82 ± 0.00** | 0.26 ± 0.00** | 0.29 ± 0.01** | 0.31 ± 0.0**1 | 0.02 ± 0.01** |
| *NtNCED3b* | 1.33 ± 0.01** | 1.00 ± 0.01 | 1.16 ± 0.03** | 0.11 ± 0.00** | 0.22 ± 0.00** | 0.28 ± 0.0**0 | 0.00 ± 0.00** |
| *NtNCED5a* | 0.27 ± 0.02 | 1.00 ± 0.02 | 0.28 ± 0.04 | 0.11 ± 0.01 | 0.08 ± 0.01 | 181.92 ± 16.32** | 0.48 ± 0.04 |
| *NtNCED5b* | 0.89 ± 0.03 | 1.00 ± 0.04 | 0.43 ± 0.05 | 35.44 ± 0.45 | 0.24 ± 0.05 | 497.28 ± 47.63** | 0.20 ± 0.02 |
| *NtNCED6a* | 0.52 ± 0.04* | 1.00 ± 0.08 | 0.27 ± 0.05** | 0.80 ± 0.03 | 0.90 ± 0.09 | 1.16 ± 0.12 | 1.17 ± 0.44 |
| *NtNCED6b* | 16.70 ± 2.36** | 1.00 ± 0.00 | 0.22 ± 0.03* | 0.14 ± 0.04* | 0.16 ± 0.05* | 4.53 ± 0.16* | 80.20 ± 3.14** |

* $p < 0.05$, ** $p < 0.01$.

**Table S4a.** The expression patterns of *NtCCDs* genes in the ABA treatment

| Gene name | 0 h | 1 h | 3 h | 6 h | 9 h | 12 h | 24 h | 48 h |
|---|---|---|---|---|---|---|---|---|
| *NtCCD1a* | 1.00 ± 0.06 | 1.80 ± 0.09** | 0.83 ± 0.01** | 1.25 ± 0.09** | 0.91 ± 0.04 | 0.34 ± 0.04** | 2.22 ± 0.09** | 1.76 ± 0.04** |
| *NtCCD1b* | 1.00 ± 0.11 | 2.10 ± 0.15** | 0.88 ± 0.06 | 0.98 ± 0.05 | 0.53 ± 0.02** | 0.32 ± 0.05** | 2.41 ± 0.10** | 2.45 ± 0.01** |
| *NtCCD4a* | 1.00 ± 0.09 | 1.99 ± 0.10** | 1.03 ± 0.11 | 4.57 ± 0.28** | 1.69 ± 0.06** | 0.32 ± 0.02** | 1.16 ± 0.16 | 1.46 ± 0.23** |
| *NtCCD4b* | 1.01 ± 0.13 | 3.13 ± 0.10** | 1.91 ± 0.03** | 12.47 ± 0.54** | 3.64 ± 0.26** | 0.67 ± 0.06 | 2.18 ± 0.01** | 3.57 ± 0.75** |
| *NtCCD4c* | 1.00 ± 0.12 | 1.75 ± 0.09** | 1.71 ± 0.13** | 3.93 ± 0.11** | 1.91 ± 0.13** | 0.40 ± 0.06** | 0.92 ± 0.08 | 1.01 ± 0.02 |
| *NtCCDLc* | 1.01 ± 0.19 | 1.36 ± 0.19 | 1.84 ± 037** | 3.10 ± 0.311** | 1.31 ± 0.13 | 0.32 ± 0.03** | 0.16 ± 0.04** | 1.72 ± 0.04** |
| *NtNCED2* | 1.01 ± 0.22 | 1.52 ± 0.01** | 1.08 ± 0.22 | 1.38 ± 0.09* | 1.51 ± 0.05** | 0.85 ± 0.02 | 3.00 ± 0.06** | 1.25 ± 0.26 |
| *NtNCED3a* | 1.01 ± 0.13 | 0.96 ± 0.04 | 2.50 ± 0.64** | 0.20 ± 0.03** | 0.53 ± 0.05* | 0.19 ± 0.01** | 0.17 ± 0.01** | 0.12 ± 0.03** |
| *NtNCED3b* | 1.00 ± 0.05 | 1.45 ± 0.08** | 3.22 ± 0.08** | 0.40 ± 0.04** | 1.07 ± 0.06 | 0.60 ± 0.07** | 0.21 ± 0.01** | 0.16 ± 0.01** |
| *NtNCED5a* | 1.01 ± 0.21 | 0.43 ± 0.01** | 0.41 ± 0.02 | 0.29 ± 0.01* | 0.31 ± 0.01 | 0.38 ± 0.04 | 0.22 ± 0.00** | 0.51 ± 0.08** |
| *NtNCED5b* | 1.00 ± 0.07 | 0.96 ± 0.04 | 0.55 ± 0.06** | 0.53 ± 0.07** | 0.43 ± 0.08** | 1.06 ± 0.12 | 0.26 ± 0.02** | 0.64 ± 0.03** |

* $p < 0.05$, ** $p < 0.01$.

**Table S4b.** The expression patterns of *NtCCDs* genes in the MeJA treatment

| Gene name | 0 h | 1 h | 3 h | 6 h | 9 h | 12 h | 24 h | 48 h |
|---|---|---|---|---|---|---|---|---|
| *NtCCD1a* | 1.00 ± 0.09 | 1.43 ± 0.20** | 0.75 ± 0.00** | 1.26 ± 0.02 | 1.23 ± 0.11 | 1.04 ± 0.05 | 1.35 ± 0.13 | 1.56 ± 0.08** |
| *NtCCD1b* | 1.00 ± 0.03 | 0.74 ± 0.10** | 0.32 ± 0.02** | 0.50 ± 0.06** | 0.18 ± 0.03** | 0.48 ± 0.04** | 0.98 ± 0.12 | 0.79 ± 0.04** |
| *NtCCD4a* | 1.00 ± 0.06 | 0.09 ± 0.01** | 0.09 ± 0.00** | 2.08 ± 0.20** | 0.48 ± 0.01** | 0.35 ± 0.02** | 1.10 ± 0.07 | 0.50 ± 0.03** |
| *NtCCD4b* | 1.00 ± 0.01 | 0.14 ± 0.02** | 0.33 ± 0.05** | 4.49 ± 0.23** | 0.59 ± 0.02** | 0.55 ± 0.04** | 1.55 ± 0.11** | 0.55 ± 0.03** |
| *NtCCD4c* | 1.00 ± 0.11 | 0.58 ± 0.08 | 0.62 ± 0.05 | 1.42 ± 0.05 | 1.44 ± 0.18 | 2.16 ± 0.11** | 1.73 ± 0.13** | 1.45 ± 0.16 |
| *NtCCD8b* | 1.02 ± 0.27 | 2.37 ± 0.29 | 4.00 ± 0.22** | 10.36 ± 2.32** | 0.04 ± 0.01 | 1.65 ± 0.41 | 0.16 ± 0.04 | 0.78 ± 0.02 |
| *NtCCDLb* | 1.00 ± 0.00 | 6.62 ± 1.16** | 4.61 ± 0.56** | 12.14 ± 1.37** | 0.17 ± 0.04 | 2.44 ± 0.24 | 0.41 ± 0.057 | 0.88 ± 0.02 |
| *NtCCDLc* | 1.00 ± 0.05 | 0.13 ± 0.01** | 0.05 ± 0.01** | 0.08 ± 0.00** | 0.13 ± 0.00** | 0.25 ± 0.01** | 0.39 ± 0.07** | 0.14 ± 0.01** |
| *NtNCED2* | 1.00 ± 0.01 | 0.13 ± 0.01** | 0.25 ± 0.00** | 2.36 ± 0.01** | 0.33 ± 0.05** | 0.24 ± 0.01** | 1.03 ± 0.07 | 0.70 ± 0.06** |
| *NtNCED3a* | 1.00 ± 0.03 | 2.92 ± 0.54** | 10.51 ± 0.37** | 6.98 ± 0.48** | 3.63 ± 0.09** | 1.69 ± 0.08* | 2.89 ± 0.19** | 2.23 ± 0.04** |
| *NtNCED3b* | 1.00 ± 0.06** | 3.13 ± 0.41** | 8.19 ± 0.42** | 8.12 ± 0.04** | 11.49 ± 0.62** | 3.66 ± 0.09** | 3.58 ± 0.41** | 1.91 ± 0.09** |
| *NtCCD5a* | 1.00 ± 0.09 | 13.92 ± 1.50** | 7.11 ± 1.39** | 11.89 ± 2.19** | 0.55 ± 0.02 | 4.10 ± 0.64** | 1.63 ± 0.05 | 2.13 ± 0.44 |
| *NtCCD5b* | 1.01 ± 0.16 | 13.73 ± 0.91** | 4.88 ± 0.16** | 5.19 ± 0.97** | 0.49 ± 0.02 | 4.64 ± 0.23** | 1.48 ± 0.20 | 1.38 ± 0.03 |

* $p < 0.05$, ** $p < 0.01$.

**Table S4c.** The expression patterns of *NtCCDs* genes in the IAA treatment

| Gene name | 0 h | 1 h | 3 h | 6 h | 9 h | 12 h | 24 h | 48 h |
|---|---|---|---|---|---|---|---|---|
| *NtCCD1a* | 1.00 ± 0.11 | 1.03 ± 0.04 | 0.99 ± 0.05 | 1.60 ± 0.05** | 0.35 ± 0.05** | 0.51 ± 0.06** | 2.71 ± 0.20** | 1.84 ± 0.28** |
| *NtCCD1b* | 1.01 ± 0.17 | 0.66 ± 0.08** | 0.55 ± 0.09** | 0.71 ± 0.03** | 0.12 ± 0.00** | 0.14 ± 0.02** | 0.68 ± 0.08** | 1.28 ± 0.07** |
| *NtCCD4a* | 1.00 ± 0.10 | 0.69 ± 0.16 | 1.16 ± 0.16 | 2.11 ± 0.30** | 0.10 ± 0.01** | 0.15 ± 0.02** | 0.33 ± 0.10** | 1.38 ± 0.19* |
| *NtCCD4b* | 1.01 ± 0.24 | 0.21 ± 0.01** | 0.56 ± 0.04** | 1.73 ± 0.03** | 0.47 ± 0.11** | 0.29 ± 0.03** | 0.58 ± 0.01** | 1.31 ± 0.15* |
| *NtCCD4c* | 1.00 ± 0.07 | 3.89 ± 0.24** | 4.70 ± 0.24** | 1.21 ± 0.05 | 9.96 ± 0.63** | 3.66 ± 0.45** | 3.15 ± 0.40** | 8.17 ± 1.20** |
| *NtCCD8b* | 1.06 ± 0.48 | 1.16 ± 0.02 | 3.56 ± 0.49 | 3.81 ± 0.13 | 1.05 ± 0.25 | 14.34 ± 3.55** | 0.53 ± 0.09 | 2.04 ± 0.03 |
| *NtCCDLc* | 1.01 ± 0.16 | 2.92 ± 0.13 | 28.62 ± 4.64** | 6.90 ± 0.25* | 39.79 ± 3.31** | 35.52 ± 1.27** | 30.38 ± 3.00** | 11.01 ± 1.66** |
| *NtNCED2* | 1.02 ± 0.24 | 1.68 ± 0.16 | 3.73 ± 0.52** | 1.69 ± 0.02 | 12.17 ± 0.42** | 9.32 ± 0.22** | 6.83 ± 0.74** | 7.52 ± 0.00** |
| *NtNCED3a* | 1.01 ± 0.15 | 1.32 ± 0.19* | 2.87 ± 0.13** | 1.60 ± 0.05** | 0.27 ± 0.07** | 0.47 ± 0.02** | 0.84 ± 0.03 | 0.73 ± 0.18* |
| *NtNCED3b* | 1.01 ± 0.16 | 0.88 ± 0.06 | 2.51 ± 0.15** | 0.83 ± 0.13 | 0.24 ± 0.02** | 0.56 ± 0.03** | 0.61 ± 0.12** | 0.54 ± 0.09** |
| *NtNCED5a* | 1.00 ± 0.06 | 5.47 ± 0.15** | 14.16 ± 1.31** | 2.71 ± 0.12** | 3.00 ± 0.06** | 3.51 ± 0.41** | 1.25 ± 0.17 | 1.95 ± 0.27 |
| *NtNCED5b* | 1.00 ± 0.06 | 6.69 ± 0.11** | 7.96 ± 1.04** | 1.92 ± 0.05* | 3.03 ± 0.01** | 4.02 ± 0.09** | 1.21 ± 0.22 | 1.50 ± 0.18 |

* $p < 0.05$, ** $p < 0.01$.

**Table S4d.** The expression patterns of *NtCCDs* genes in the SA treatment

| Gene Name | 0 h | 1 h | 3 h | 6 h | 9 h | 12 h | 24 h | 48 h |
|---|---|---|---|---|---|---|---|---|
| *NtCCD1a* | 1.00 ± 0.07 | 1.23 ± 0.14 | 2.24 ± 0.12** | 1.54 ± 0.18** | 1.67 ± 0.23* | 2.49 ± 0.50** | 0.66 ± 0.11 | 0.68 ± 0.04 |
| *NtCCD1b* | 1.00 ± 0.03 | 0.46 ± 0.05** | 0.41 ± 0.00** | 0.18 ± 0.02** | 1.34 ± 0.09** | 0.12 ± 0.03** | 0.40 ± 0.04** | 0.32 ± 0.06** |
| *NtCCD4a* | 1.04 ± 0.39 | 0.36 ± 0.06** | 2.50 ± 0.10** | 1.41 ± 0.08* | 2.27 ± 0.13** | 0.33 ± 0.09** | 0.10 ± 0.06** | 0.14 ± 0.01** |
| *NtCCD4b* | 1.02 ± 0. 23 | 1.03 ± 0.06 | 4.44 ± 0.24** | 8.47 ± 0.95** | 3.44 ± 0.28** | 1.75 ± 0.45* | 0.61 ± 0.10 | 0.71 ± 0.10 |
| *NtCCD4c* | 0.99 ± 0.09 | 0.81 ± 0.12 | 0.91 ± 0.01 | 1.82 ± 0.14** | 0.82 ± 0.02 | 0.91 ± 0.14 | 1.02 ± 0.03 | 1.09 ± 0.05 |
| *NtCCDLc* | 1.02 ± 0.07 | 2.11 ± 0.60 | 8.01 ± 0.43 | 24.93 ± 0.24** | 22.07 ± 2.07** | 69.78 ± 10.46** | 41.31 ± 5.51** | 27.05 ± 3.96** |
| *NtNCED2* | 1.00 ± 0.09 | 0.52 ± 0.04 | 1.04 ± 0.06 | 1.23 ± 1.07 | 0.45 ± 0.02 | 1.06 ± 0.11 | 1.51 ± 0.04 | 0.79 ± 0.06 |
| *NtNCED3a* | 1.00 ± 0.06 | 0.44 ± 0.08* | 0.14 ± 0.00** | 0.16 ± 0.01** | 2.76 ± 0.43** | 0.65 ± 0.11 | 0.12 ± 0.00** | 3.11 ± 0.11** |
| *NtNCED3b* | 1.00 ± 0.07 | 1.59 ± 0.04** | 0.46 ± 0.10* | 0.25 ± 0.03** | 0.78 ± 0.04 | 2.51 ± 0.49** | 0.83 ± 0.16 | 1.76 ± 0.29** |
| *NtNCED5a* | 0.98 ± 0.11 | 0.67 ± 0.12** | 2.38 ± 0.11** | 0.84 ± 0.05 | 0.28 ± 0.01** | 0.20 ± 0.05** | 0.92 ± 0.06 | 0.33 ± 0.02** |
| *NtNCED5b* | 0.99 ± 0.02 | 1.28 ± 0.08 | 5.54 ± 0.69** | 1.88 ± 0.18** | 0.39 ± 0.01 | 0.58 ± 0.03 | 1.10 ± 0.03 | 0.57 ± 0.02 |

* $p < 0.05$, ** $p < 0.01$.

**Table S4e.** The expression patterns of *NtCCDs* genes in the drought stress

| Gene name | 0 h | 1 h | 3 h | 6 h | 9 h | 12 h | 24 h | 48 h |
|---|---|---|---|---|---|---|---|---|
| *NtCCD1a* | 1.00 ± 0.10 | 0.82 ± 0.05* | 0.75 ± 0.05** | 0.81 ± 0.07* | 0.62 ± 0.04** | 1.08 ± 0.07 | 2.26 ± 0.01** | 2.64 ± 0.21** |
| *NtCCD1b* | 1.00 ± 0.03 | 0.69 ± 0.04** | 0.62 ± 0.02** | 0.40 ± 0.01** | 0.23 ± 0.01** | 0.18 ± 0.01** | 1.00 ± 0.02 | 0.76 ± 0.04** |
| *NtCCD4a* | 1.00 ± 0.11 | 1.91 ± 0.10** | 0.98 ± 0.07 | 0.52 ± 0.01** | 0.30 ± 0.05** | 0.16 ± 0.01** | 1.36 ± 0.06** | 0.23 ± 0.02** |
| *NtCCD4b* | 1.00 ± 0.04 | 2.51 ± 0.18** | 3.15 ± 0.10** | 1.71 ± 0.15** | 1.05 ± 0.03 | 0.38 ± 0.04** | 0.57 ± 0.01** | 0.30 ± 0.03** |
| *NtCCD4c* | 1.00 ± 0.08 | 1.49 ± 0.08** | 2.30 ± 0.02** | 2.54 ± 0.05** | 0.94 ± 0.07 | 0.41 ± 0.01** | 5.10 ± 0.07** | 0.98 ± 0.01 |
| *NtCCD8b* | 1.00 ± 0.04 | 5.77 ± 0.21** | 2.42 ± 0.70 | 5.29 ± 1.61** | 5.86 ± 0.80** | 8.79 ± 0.52** | 4.73 ± 0.44 | 9.20 ± 1.97** |
| *NtCCDLc* | 1.03 ± 0.32 | 4.33 ± 0.17** | 8.71 ± 0.26** | 2.52 ± 0.10** | 2.86 ± 0.49** | 1.45 ± 0.07 | 3.93 ± 0.19** | 0.51 ± 0.06 |
| *NtNCED2* | 1.01 ± 0.18 | 6.05 ± 0.03** | 6.94 ± 0.53** | 3.68 ± 0.45** | 2.64 ± 0.11** | 2.93 ± 0.06** | 2.93 ± 0.30** | 1.14 ± 0.19 |
| *NtNCED3a* | 1.00 ± 0.08 | 3.55 ± 0.27** | 0.79 ± 0.11 | 0.68 ± 0.06 | 0.55 ± 0.00* | 0.46 ± 0.01** | 7.40 ± 0.14** | 3.75 ± 0.43** |
| *NtNCED3b* | 1.01 ± 0.16 | 3.69 ± 0.34** | 0.63 ± 0.01 | 1.44 ± 0.04* | 1.03 ± 0.15 | 1.20 ± 0.18 | 4.86 ± 0.02** | 3.64 ± 0.26** |
| *NtNCED5a* | 1.00 ± 0.12 | 6.39 ± 0.39** | 10.46 ± 0.30** | 34.59 ± 2.71** | 8.69 ± 1.17** | 1.93 ± 0.32 | 9.58 ± 0.09** | 39.72 ± 2.72** |
| *NtNCED5b* | 1.00 ± 0.05 | 0.26 ± 0.03* | 0.32 ± 0.05* | 2.32 ± 0.73** | 0.18 ± 0.04* | 0.03 ± 0.00** | 0.20 ± 0.01* | 0.14 ± 0.01* |

* $p < 0.05$, ** $p < 0.01$.

**Table S4f.** The expression patterns of *NtCCDs* genes in the cold stress

| Gene name | 0 h | 1 h | 3 h | 6 h | 9 h | 12 h | 24 h | 48 h |
|---|---|---|---|---|---|---|---|---|
| *NtCCD1a* | 1.00 ± 0.04 | 1.15 ± 0.02** | 1.35 ± 0.10** | 1.16 ± 0.06** | 1.41 ± 0.03** | 1.29 ± 0.02** | 0.82 ± 0.02** | 0.94 ± 0.02 |
| *NtCCD1b* | 1.00 ± 0.05 | 0.73 ± 0.03** | 0.77 ± 0.04** | 0.76 ± 0.04** | 1.33 ± 0.05** | 0.84 ± 0.02** | 0.84 ± 0.10** | 0.52 ± 0.01** |
| *NtCCD4a* | 1.00 ± 0.03 | 2.20 ± 0.05** | 1.51 ± 0.13** | 1.39 ± 0.03** | 1.52 ± 0.09** | 1.66 ± 0.05** | 1.35 ± 0.13** | 0.45 ± 0.03** |
| *NtCCD4b* | 1.00 ± 0.04 | 2.10 ± 0.15** | 1.09 ± 0.12 | 1.38 ± 0.08** | 1.19 ± 0.05 | 2.61 ± 0.06** | 1.69 ± 0.19** | 0.99 ± 0.03 |
| *NtCCD4c* | 1.01 ± 0.21 | 1.34 ± 0.08* | 1.53 ± 0.21** | 1.23 ± 0.02 | 1.55 ± 0.16** | 1.66 ± 0.17** | 1.05 ± 0.08 | 0.39 ± 0.04** |
| *NtNCED2* | 1.01 ± 0.16 | 1.12 ± 0.08 | 1.90 ± 0.07** | 0.82 ± 0.07 | 1.52 ± 0.11** | 1.64 ± 0.01** | 2.62 ± 0.17** | 0.74 ± 0.05* |
| *NtNCED3a* | 1.00 ± 0.04 | 0.88 ± 0.05 | 1.70 ± 0.17** | 1.27 ± 0.16* | 1.68 ± 0.11** | 2.16 ± 0.19** | 4.09 ± 0.12** | 1.83 ± 0.15** |
| *NtNCED3b* | 1.00 ± 0.08 | 1.52 ± 0.03 | 3.21 ± 0.26** | 2.17 ± 0.12** | 2.63 ± 0.11** | 3.20 ± 0.49** | 6.73 ± 0.86** | 2.23 ± 0.22** |
| *NtNCED5a* | 1.04 ± 0.43 | 4.71 ± 0.22 | 18.94 ± 0.20** | 34.01 ± 1.58** | 48.09 ± 4.48** | 29.44 ± 2.34** | 6.96 ± 0.07* | 13.41 ± 2.74** |
| *NtNCED5b* | 1.01 ± 0.20 | 5.31 ± 0.06* | 21.05 ± 0.92** | 29.63 ± 0.93** | 57.34 ± 3.65** | 33.19 ± 3.09** | 6.66 ± 0.54** | 8.09 ± 0.55** |

* $p < 0.05$, ** $p < 0.01$.

**Table S4g.** The expression patterns of *NtCCDs* genes in the heat stress

| Gene name | 0 h | 1 h | 3 h | 6 h | 9 h | 12 h | 24 h | 48 h |
|---|---|---|---|---|---|---|---|---|
| *NtCCD1a* | 1.01 ± 0.22 | 0.39 ± 0.11** | 0.43 ± 0.12** | 0.45 ± 0.13** | 0.32 ± 0.14** | 0.61 ± 0.14** | 0.42 ± 0.14** | 0.31 ± 0.18** |
| *NtCCD1b* | 1.00 ± 0.11 | 0.47 ± 0.02** | 0.49 ± 0.04** | 0.26 ± 0.02** | 0.14 ± 0.01** | 0.25 ± 0.01** | 0.64 ± 0.04** | 0.38 ± 0.03** |
| *NtCCD4a* | 1.01 ± 0.14 | 0.42 ± 0.03** | 0.61 ± 0.01** | 0.80 ± 0.11** | 0.27 ± 0.03** | 0.25 ± 0.00** | 0.03 ± 0.00** | 0.01 ± 0.00** |
| *NtCCD4b* | 1.01 ± 0.14 | 0.17 ± 0.01** | 0.86 ± 0.03** | 0.91 ± 0.05 | 0.24 ± 0.01** | 0.23 ± 0.02** | 0.03 ± 0.00** | 0.04 ± 0.00** |
| *NtCCD4c* | 1.02 ± 0.23 | 0.16 ± 0.02** | 0.28 ± 0.01** | 4.03 ± 0.20** | 0.26 ± 0.05** | 0.19 ± 0.03** | 1.44 ± 0.06** | 0.70 ± 0.00** |
| *NtNCED2* | 1.00 ± 0.03 | 0.80 ± 0.02 | 1.43 ± 0.26** | 2.42 ± 0.10** | 1.93 ± 0.05** | 0.96 ± 0.13 | 3.68 ± 0.15** | 1.01 ± 0.00 |
| *NtNCED3a* | 1.01 ± 0.15 | 0.20 ± 0.02** | 0.61 ± 0.03** | 0.18 ± 0.01** | 0.96 ± 0.04 | 0.78 ± 0.02** | 1.22 ± 0.04** | 0.30 ± 0.00** |
| *NtNCED3b* | 1.00 ± 0.11 | 0.13 ± 0.02** | 0.53 ± 0.02** | 0.19 ± 0.02** | 0.53 ± 0.01** | 0.75 ± 0.03** | 0.44 ± 0.01** | 0.10 ± 0.01** |
| *NtNCED5a* | 1.00 ± 0.07 | 0.50 ± 0.04 | 1.82 ± 0.03** | 2.61 ± 0.09** | 6.65 ± 0.91** | 0.40 ± 0.04* | 1.69 ± 0.06* | 1.25 ± 0.05 |
| *NtNCED5b* | 1.01 ± 0.18 | 0.98 ± 0.12 | 2.50 ± 0.31 | 2.36 ± 0.20 | 16.26 ± 4.48** | 0.87 ± 0.14 | 2.19 ± 0.42 | 2.31 ± 0.08 |

*, $p < 0.05$, **, $p < 0.01$.

**Table S5** The primer of *NtCCDs*

| | F | R |
|---|---|---|
| *NtCCD1a-RT* | TGGGAAGGCCATAGACTTATT | AGAGGAGGAGTTTCATCAGTAG |
| *NtCCD1b-RT* | CTGATGCACGACTCCAGTA | CGGAAGATGGCCTTTAACAG |
| *NtCCD4a-RT* | GCCACAAACCACTACTAGAAC | GATGGAAATGATGGCTTTACTG |
| *NtCCD4b-RT* | AGAAAGGCCACAAACAACT | CTGATGGAAACGATGGCTC |
| *NtCCD4c-RT* | TACCACTACCTCGCATTCAG | ACCATTTCCATCGGTTATCT |
| *NtCCD7a-RT* | ATCCCACTGAGGGTTTAGGT | CAATGATGAATGTAGAAGGAGGAG |
| *NtCCD7b-RT* | CCTGACCTGAACCACCATCT | ACTGAACCCATCACCCTTCG |
| *NtCCD8a-RT* | GGCTTAGGTACTTCGGAAAA | AGAAATGGCCCAGGACAATG |
| *NtCCD8b-RT* | TCGCGTTATCGGTTAGGGAC | AGGCAAATCGAATCGGAGGC |
| *NtCCDLa-RT* | ACAGAGGATTTGAGCAGGAG | ATTTGTTGACCAGCCATTAC |
| *NtCCDLb-RT* | TGACAACCCAACCATCATTC | GGGCTTGCCAAACTTCATTT |
| *NtCCDLc-RT* | TTTCTAGGGAATGTATGTCG | TCTTACTGGCGTACCTTCTA |
| *NtNCED2-RT* | TCCCGAACCTAGATTTCTTCTTCT | CGTTGTGATCCCTGACCAGC |
| *NtNCED3a-RT* | CCCAAGATATCAATGCCAAGAG | AAATGGAGAATTGGAGGAGC |
| *NtNCED3b-RT* | CCATCTTCAAGAGACTTGGC | AAATGGAGAATTGGAGGAGC |
| *NtNCED5a-RT* | TTTGAACTTGATACGGGACA | CTTCTACGCTCGTGGACTAT |
| *NtNCED5b-RT* | CTGTAACCGGAAGACTATGC | TCAGCCTCAAACTCCTGACT |
| *NtNCED6a-RT* | GATTCGTCGTGATCGTCTTT | TTTCTATTTCCCTCCAACATC |
| *NtNCED6b-RT* | TCATACCCGATACTATAACCC | AGATAAACGAAGACGACGAT |
